# Exploring the Link Between Additive Heritability and Prediction Accuracy From a Ridge Regression Perspective

**DOI:** 10.3389/fgene.2020.581594

**Published:** 2020-11-04

**Authors:** Arthur Frouin, Claire Dandine-Roulland, Morgane Pierre-Jean, Jean-François Deleuze, Christophe Ambroise, Edith Le Floch

**Affiliations:** ^1^CNRGH, Institut Jacob, CEA - Université Paris-Saclay, Évry, France; ^2^Centre d'Etude du Polymorphisme Humain, Fondation Jean Dausset, Paris, France; ^3^LaMME, Université Paris-Saclay, CNRS, Université d'Évry val d'Essonne, Évry, France

**Keywords:** heritability, prediction accuracy, ridge regression, mixed model, generalized cross validation, best linear unbiased predictor

## Abstract

Genome-Wide Association Studies (GWAS) explain only a small fraction of heritability for most complex human phenotypes. Genomic heritability estimates the variance explained by the SNPs on the whole genome using mixed models and accounts for the many small contributions of SNPs in the explanation of a phenotype. This paper approaches heritability from a machine learning perspective, and examines the close link between mixed models and ridge regression. Our contribution is two-fold. First, we propose estimating genomic heritability using a predictive approach via ridge regression and Generalized Cross Validation (GCV). We show that this is consistent with classical mixed model based estimation. Second, we derive simple formulae that express prediction accuracy as a function of the ratio $\frac{n}{p}$, where *n* is the population size and *p* the total number of SNPs. These formulae clearly show that a high heritability does not imply an accurate prediction when *p* > *n*. Both the estimation of heritability via GCV and the prediction accuracy formulae are validated using simulated data and real data from UK Biobank.

## 1. Introduction

The old nature vs. nurture debate is about whether a complex human trait is determined by a person's genes or by the environment. It is a longstanding philosophical question that has been reinvestigated in the light of statistical genetics (Feldman and Lewontin, 1975). The concept of heritability was introduced by Fisher (1918) and Wright (1920, 1921) in the context of pedigree data. It has proved highly useful in animal (Meuwissen et al., 2001) and plant genetics (Xu, 2003) for selection purposes because of its association with accurate prediction of a trait from genetic data. In the last decades, Genome-Wide Association Studies (GWAS) have become highly popular for identifying variants associated with complex human traits (Hirschhorn and Daly, 2005). They have recently been used for heritability estimations (Yang et al., 2010). A shortcut is often made between the heritability of a trait and the prediction of this trait. However, heritable complex human traits are often caused by a large number of genetic variants that individually make small contributions to the trait variation, which is often referred to as polygeny. In this context, the relation between heritability and prediction accuracy may not hold (de Vlaming and Groenen, 2015).

The goal of this paper is to establish a clear relation between prediction accuracy and heritability, especially when the number of genetic markers is much higher than the population size, which is typically the case in GWAS. Based on the linear model, statistical analyses of SNP data address very different and sometimes unrelated questions. The most commonly performed analyses tend to be association studies, where multiple hypothesis testing makes it possible to test the link between any SNP and a phenotype of interest. In genomic selection, markers are selected to predict a phenotype with a view to selecting an individual in a breeding population. Association studies and genomic selection may identify different sets of markers, since even weak associations might be of interest for prediction purposes, while not all strongly associated markers are necessarily useful, because of redundancy through linkage disequilibrium. Genomic heritability allows quantifying the amount of genomic information relative to a given phenotype via mixed model parameter estimation. The prediction of the phenotype using all genomic information via the mixed model is a closely related but different problem.

We approach the problem of heritability estimation from a machine learning perspective. This is not a classical approach in genetics, where inferential statistics is the usual preferred tool. In this context, heritability is considered as a parameter to be inferred from a small sample of the population. The machine learning approach places the emphasis on prediction accuracy. It makes a clear distinction between performance on training samples and performance on testing samples, whereas inferential statistics focuses on parameter estimation on a single dataset.

### 1.1. Classical Approach via Mixed Models

Heritability is defined as the proportion of phenotypic variance due to genetic factors. A quantitative definition of heritability requires a statistical model. The model commonly adopted is a simple three-term model without gene-environment interaction (Henderson, 1975):

$$\begin{array}{l} \text{y}=\text{g}+\text{f}+\text{e}, \end{array}$$

where **y** ∈ ℝ^*n*^ is a quantitative phenotype vector describing *n* individuals, **f** ∈ ℝ^*n*^ is a non-genetic covariate term, **g** ∈ ℝ^*n*^ is a genetic term and **e** ∈ ℝ^*n*^ an environmental residual term. The term **g** will depend on the diploid genotype matrix $\text{M}\in M_{n,p}\left(\mathbb{R}\right)$ of the *p* causal variants.

There are two definitions of heritability in common use: first, there is H^2^, heritability in the broad sense, measuring the overall contribution of the genome; and second, there is *h*^2^, heritability in the narrow sense (also known as additive heritability), defined as the proportion of phenotypic variance explained by the additive effects of variants.

The quantification of narrow-sense heritability goes back to family studies by Fisher (1918), who introduced the above model with the additional hypothesis that **g** is the sum of independent genetic terms, and with **e** assumed to be normal. This heritability in the narrow sense is a function of the correlation between the phenotypes of relatives.

Although Fisher's original model makes use of pedigrees for parameter estimation, some geneticists have proposed using the same model with genetic data from unrelated individuals (Yang et al., 2011a).

#### 1.1.1. Polygenic Model

In this paper, we focus on the version of the additive polygenic model with a Gaussian noise where **g** = **Z***u*, **f** = **X**β, with $\text{Z}\in M_{n,p}\left(\mathbb{R}\right)$ a standardized (by columns) version of **M**, *u* ∈ ℝ^*p*^ a vector of genetic effects, $\text{X}\in M_{n,r}\left(\mathbb{R}\right)$ a matrix of covariates, β ∈ ℝ^*r*^ a vector of covariate effects, μ an intercept and $\text{e}\sim N\left({\text{0}}_{n},\sigma^{2}{\text{I}}_{n}\right)$ a vector of environmental effects.

The model thus becomes

(1) $$\begin{array}{l} \text{y}=\mu {𝟙}_{n}+\text{Z}u+\text{X}\beta +\text{e}, \end{array}$$

where ${𝟙}_{n}\in {\mathbb{R}}^{n}$ a vector of ones.

#### 1.1.2. Estimation of Heritability From GWAS Results

To estimate heritability in a GWAS context, a first intuitive approach would be to estimate *u* with a least squares regression to solve problem (1). Unfortunately, this is complicated in practice for three reasons: the causal variants are not usually available among genotyped variants; genotyped variants are in linkage disequilibrium (LD); and the least squares estimate is only defined when *n* > *p*, which is not often the case in a GWAS (Yang et al., 2010).

One technique for obtaining a solvable problem is to use the classical GWAS approach to determine a subset of variants significantly associated with the phenotype. The additive heritability can then be estimated by summing their effects estimated by simple linear regressions. In practice this estimation tends to greatly underestimate *h*^2^ (Manolio et al., 2009). It only takes into account variants that have passed the significance threshold after correction for multiple comparisons (strong effects) and does not capture the variants that are weakly associated with the phenotype (weak effects).

#### 1.1.3. Estimating Heritability via the Variance of the Effects

Yang et al. (2010) suggest that most of the missing heritability comes from variants with small effects. In order to be able to estimate the information carried by weak effects they assume a linear mixed model where the vector of random genetic effects follows a normal homoscedastic distribution $u\sim N\left(0_{p},\tau {\text{I}}_{p}\right)$. They propose estimating the variance components τ and σ^2^, and defining genomic heritability as $h_{G}^{2}=\frac{p\tau }{p\tau +\sigma^{2}}$. An example of an algorithm for estimating variance components is the Average Information—Restricted Maximum Likelihood (AI-REML) algorithm, implemented in software such as Genome-wide Complex Trait Analysis (GCTA) (Yang et al., 2011a) or gaston (Perdry and Dandine-Roulland, 2018). More recent methods that are much faster than REML have also been proposed, such as the modified Haseman-Elston regression (Chen, 2014) or methods based on summary statistics such as the LD-score regression (Bulik-Sullivan et al., 2015) or the MQS (MinQue for Summary statistics) approach (Zhou, 2017).

### 1.2. A Statistical Learning Approach via Ridge Regression

The linear model is used in statistical genetics for exploring and summarizing the relation between a phenotype and one or more genetic variants, and it is also used in predictive medicine and genomic selection for prediction purposes. When used for prediction, the criterion for assessing performance is the prediction accuracy.

Although least squares linear regression is the baseline method for quantitative phenotype prediction, it has some limitations. As mentioned earlier, the estimator is not defined when the number of descriptive variables *p* is greater than the number of individuals *n*. Even when *n* > *p*, the estimator may be highly variable when the descriptive variables are correlated, which is clearly the case in genetics.

Ridge regression is a penalized version of least squares that can overcome these limitations (Hoerl and Kennard, 1970). Ridge regression is strongly related to the mixed model and is prediction-oriented.

#### 1.2.1. Ridge Regression

The ridge criterion builds on the least squares criterion, adding an extra penalization term. The penalization term is proportional to the ℓ_2_ norm of the parameter vector. The proportionality coefficient λ is also called the penalization parameter. The penalty tends to shrink the coefficients of the least squares estimator, but never cancels them out. The degree of shrinkage is controlled by λ: the higher the value of λ, the greater the shrinkage:

(2) $$\begin{array}{l} {\hat{u}}_{R}=\underset{u}{\text{arg min }}\|\text{y}-\text{Z}u{\|}_{2}^{2}+\lambda \|u{\|}_{2}^{2}, \end{array}$$

(3) $$\begin{array}{l} ={\left({\text{Z}}^{T}\text{Z}+\lambda {\text{I}}_{p}\right)}^{-1}{\text{Z}}^{T}\text{y}, \end{array}$$

(4) $$\begin{array}{l} ={\text{Z}}^{T}{\left(\text{Z}{\text{Z}}^{T}+\lambda {\text{I}}_{n}\right)}^{-1}\text{y}. \end{array}$$

Ridge regression can be seen as a *Bayesian Maximum a Posteriori* estimation of the linear regression parameters considering a Gaussian prior with hyperparameter λ.

The estimator depends on a λ that needs to be chosen. In a machine learning framework, a classical procedure is to choose the λ that minimizes the squared loss over new observations.

The practical effect of the penalty term is to add a constant to the diagonal of the covariance matrix, which makes the matrix non-singular, even in the case where *p* > *n*. When the descriptive variables are highly correlated, this improves the conditioning of the **Z**^*T*^**Z** matrix, while reducing the variance of the estimator.

The existence theorem states that there always exists a value of λ > 0 such that the Mean Square Error (MSE) of the ridge regression estimator (variance plus the squared bias) is smaller than the MSE of the Maximum Likelihood estimator (Hoerl and Kennard, 1970). This is because there is always an advantageous bias-variance compromise that reduces the variance without greatly increasing the bias.

Ridge regression also allows us to simultaneously estimate all the additive effects of the genetic variants without discarding any, which reflects the idea that all the variants make a small contribution.

#### 1.2.2. Link Between Mixed Model and Ridge Regression

This paper builds on the parallel between BLUPs (Best Linear Unbiased Predictions) derived from the mixed model and ridge regression (Meuwissen et al., 2001). The use of ridge regression in quantitative genetics has already been discussed (De los Campos et al., 2013; de Vlaming and Groenen, 2015) We look at a machine-learning oriented paradigm for estimating the ridge penalty parameter, which provides us with a direct link to heritability. There is an equivalence between maximizing the posterior *p*(*u*|**y**) and minimizing a ridge criterion (Bishop, 2006) under the assumptions that $u\sim N\left(0_{p},\tau {\text{I}}_{p}\right)$ and $\text{e}\sim N\left(0_{n},\sigma^{2}{\text{I}}_{n}\right)$ (see section 6 in Supplementary Material for details). The optimal penalty hyperparameter of the ridge criterion λ can be used to estimate the heritability. It is indeed defined as the ratio of the variance parameters of the mixed model:

(5) $$\begin{array}{l} \underset{u}{\text{arg max }}p\left(u|\text{y}\right)=\underset{u}{\text{arg min }}\|\text{y}-\text{Z}u{\|}_{2}^{2}+\lambda \|u{\|}_{2}^{2}\text{with}\lambda =\frac{\sigma^{2}}{\tau }. \end{array}$$

The relation between λ and $h_{G}^{2}$ (de Vlaming and Groenen, 2015) is thus:

(6) $$\begin{array}{l} h_{G}^{2}=\frac{p}{p+\lambda };\lambda =p\frac{1-h_{G}^{2}}{h_{G}^{2}}. \end{array}$$

Consequently, the BLUP has a similar formulation to the ridge estimator. Indeed, as shown in the section 6.2 of the article by Robinson et al. (1991), its general definition is:

(7) $$\begin{array}{l} {\hat{u}}_{BLUP}=\hat{𝔼\left(u|\text{y}\right)}=\hat{\text{W}}{\text{Z}}^{T}{\hat{\Sigma }}^{-1}\left(\text{y}-\text{X}\hat{\beta }\right), \end{array}$$

where $u\sim N\left(0_{p},\text{W}\right)$, $\text{e}\sim N\left(0_{n},\text{E}\right)$ and **Σ** = **ZWZ**^*T*^ + **E**.

When we further assume β = 0_*r*_, **W** = τ**I**_*p*_ and $\text{E}=\sigma^{2}{\text{I}}_{n}$, it becomes:

(8) $$\begin{array}{l} {\hat{u}}_{BLUP}=\tau {\text{Z}}^{T}{\left(\tau \text{Z}{\text{Z}}^{T}+\sigma^{2}{\text{I}}_{n}\right)}^{-1}\text{y}={\text{Z}}^{T}{\left(\text{Z}{\text{Z}}^{T}+\frac{\sigma^{2}}{\tau }{\text{I}}_{n}\right)}^{-1}\text{y}, \end{array}$$

which is exactly the ridge estimator.

#### 1.2.3. Over-Fitting

Interestingly, ridge regression and the mixed model can be seen as two similar ways to deal with the classical over-fitting issue in machine learning, which is where a learner becomes overspecialized in the dataset used for the estimation of its parameters and is unable to generalize (Bishop, 2006). When *n* > *p*, estimating the parameters of a fixed-effect linear model via maximum likelihood estimation may lead to over-fitting, when too many variables are considered. A classical way of reducing over-fitting is regularization, and in order to set the value of the regularization parameter there are two commonly adopted approaches: first, the Bayesian approach, and second, the use of additional data.

Mixed Model parameter estimation via maximum likelihood can be seen as a type of self-regularizing approach (see Equation 5). Estimating the variance components of the mixed model may be interpreted as a kind of empirical Bayes approach, where the ratio of the variances is the regularization parameter that is usually estimated using a single dataset. In contrast to this, in order to properly estimate the ridge regression regularization hyperparameter that gives the best prediction, two datasets are required. If a single dataset were to be used, this would result in an insufficiently regularized (i.e., excessively complex) model offering too high prediction performances on the present dataset but unable to predict new samples well. This over-fitting phenomenon is particularly evident when dimensionality is high.

The fact that the complexity of the ridge model is controlled by its hyperparameter can be intuitively understood when considering extreme situations. When λ tends to infinity, the estimated effect vector (i.e., $\hat{u}$_*R*_) tends to the null vector. Conversely, when λ tends to zero, the model approaches maximum complexity. One solution for choosing the right complexity is therefore to use both a training set to estimate the effect vector for different values of the hyperparameter and a validation set to choose the hyperparameter value with the best prediction capacity on this independent sample. An alternative solution, when data is sparse, is to use a cross-validation approach to mimic a two-set situation. Finally, it should be noted that the estimation of prediction performance on a validation dataset is still overoptimistic, and consequently a third dataset, known as a test set, is required to assess the real performance of the model.

#### 1.2.4. Prediction Accuracy in Genetics

In genomic selection and in genomic medicine, several authors have been interested in predicting complex traits that show a relatively high heritability using mixed model BLUPs (Speed and Balding, 2014). The literature defined the prediction accuracy as the correlation between the trait and its prediction, which is unusual in machine learning where the expected loss is often preferred. Several approximations of this correlation have been proposed in the literature (Brard and Ricard, 2015), either in a low-dimensional context (where the number of variants is lower than the number of individuals) or in a high-dimensional context.

Daetwyler et al. (2008) derived equations for predicting the accuracy of a genome-wide approach based on simple least-squares regressions for continuous and dichotomous traits. They consider one univariate linear regression per variant (with a fixed effect) and combine them afterwards, which is equivalent to a Polygenic Risk Score (PRS) (Pharoah et al., 2002; Purcell et al., 2009). Goddard (2009) extended this prediction to Genomic BLUP (GBLUP), which used the concept of an effective number of loci. Rabier et al. (2016) proposed an alternative correlation formula conditionally on a given training set. Their formula refines the formula proposed by Daetwyler et al. (2008). Elsen (2017) used a Taylor development to derive the same formula in small dimension.

Using intensive simulation studies, de Vlaming and Groenen (2015) showed a strong link between PRS and ridge regression in terms of prediction accuracy, when the population size is limited. However, with ridge regression, predictive accuracy improves substantially as the sample size increases.

It is important to note a difference in the prediction accuracy of GBLUP when dealing with human populations as opposed to breeding populations (De los Campos et al., 2013). De los Campos et al. (2013) show that the squared correlation between GBLUP and the phenotype reaches the trait heritability, asymptotically when considering unrelated human subjects. Dandine-Roulland and Perdry (2015) also proposed a theoretical formula of the performance of BLUPs for prediction in the context of human genetics, which is proportional to the number of individuals, to the squared heritability and to the variance of the off-diagonal terms of the Genetic Relatedness Matrix.

Zhao and Zhu (2019) studied cross trait prediction in high dimension. They derive generic formulae for in and out-of sample squared correlation. They link the marginal estimator to the ridge estimator and to GBLUP. Their results are very generic and generalize formulae proposed by Daetwyler et al. (2008).

#### 1.2.5. Outline of the Paper

While some authors have proposed making use of the equivalence between ridge regression and the mixed model for setting the hyperparameter of ridge regression according to the heritability estimated by the mixed model, we propose on the contrary to estimate the optimal ridge hyperparameter using a predictive approach via Generalized Cross Validation. We derive approximations of the squared correlation and of the expected loss, both in high and low dimensions.

Using synthetic data and real data from UK Biobank, we show that our results are consistent with classical mixed model based estimation and that our approximations are valid.

Finally, with reference to the ridge regression estimation of heritability, we discuss how heritability is linked to prediction accuracy in highly polygenic contexts.

## 2. Materials and Methods

### 2.1. Generalized Cross Validation for Speeding Up Heritability Estimation via Ridge Regression

#### 2.1.1. Generalized Cross Validation

A classical strategy for choosing the ridge regression hyperparameter uses a grid search and *k*-fold cross validation. Each grid value of the hyperparameter is evaluated by the cross validated error. This approach is time-consuming in high dimension, since each grid value requires *k* estimations. In the machine learning context, we propose using Generalized Cross Validation (GCV) to speed up the estimation of the hyperparameter λ and thus to estimate the additive heritability $h_{G}^{2}$ using the link described in Equation (6).

The GCV error in Equation (9) (Golub et al., 1978) is an approximation of the Leave-One-Out error (LOO) (see section 2 in Supplementary Material). Unlike the classical LOO, GCV does not require *n* ridge regression estimations (where *n* is the number of observations) at each grid value, but involves a single run. It thus provides a much faster and convenient alternative for choosing the hyperparameter. We have

(9) $$\begin{array}{l} {\text{err}}^{GCV}=\frac{\|\text{y}-\hat{\text{y}}\left(\lambda \right){\|}_{2}^{2}}{{\left[\frac{1}{n}\text{tr}\left({\text{I}}_{n}-{\text{H}}_{\lambda }\right)\right]}^{2}}, \end{array}$$

where $\hat{\text{y}}\left(\lambda \right)=\text{Z}{\hat{u}}_{R}\left(\lambda \right)={\text{H}}_{\lambda }\text{y}$ is the prediction of the training set phenotypes using the same training set for the estimation of $\hat{u}$_*R*_ and where **H**_λ_ is defined as (see section 1 in Supplementary Material for details):

$$\begin{array}{l} {\text{H}}_{\lambda }=\text{Z}{\left({\text{Z}}^{T}\text{Z}+\lambda {\text{I}}_{p}\right)}^{-1}{\text{Z}}^{T}\\ \;=\text{Z}{\text{Z}}^{T}{\left(\text{Z}{\text{Z}}^{T}+\lambda {\text{I}}_{n}\right)}^{-1}. \end{array}$$

A Singular Value Decomposition (SVD) of the **H**_λ_ can be used advantageously to speed up GCV computation (see section 3 in Supplementary Material).

#### 2.1.2. Empirical Centering Can Lead to Issues in the Choice of Penalization Parameter in a High-Dimensional Setting

In high dimensional settings (*p* > *n*), the use of GCV after empirical centering of the data can lead to a strong bias in the choice of λ and thus in heritability estimation. Let us illustrate the problem with a simple simulation. We simulate a phenotype from synthetic genotype data with a known heritability of *h*^2^ = 0.25, *n* = 1, 000 individuals, *p* = 10, 000 variants and 100% causal variants. The simulation follows the additive polygenic model without intercept or covariates, as described in section 2.3. Before applying GCV, genotypes are standardized in the most naive way: the genotype matrix **M** is empirically centered and scaled column-wise, resulting in the matrix **Z**. Since we want to mimic an analysis on real data, let us assume that there is a potential intercept in our model (in practice the empirical mean of our simulated phenotype is likely to be non-null):

(10) $$\begin{array}{l} \text{y}=\mu {𝟙}_{n}+\text{Z}u+\text{e}. \end{array}$$

GCV expects all the variables to be penalized, but penalizing the intercept is not relevant. We therefore consider a natural two-step procedure: first the model's intercept is estimated via the empirical mean of the phenotype $\hat{\mu }=\frac{1}{n}\underset{i}{\sum }y_{i}$, and, second, GCV is applied on the empirically centered phenotype $\text{y}=\text{y}-\hat{\mu }{𝟙}_{n}$.

**Figure 1:**
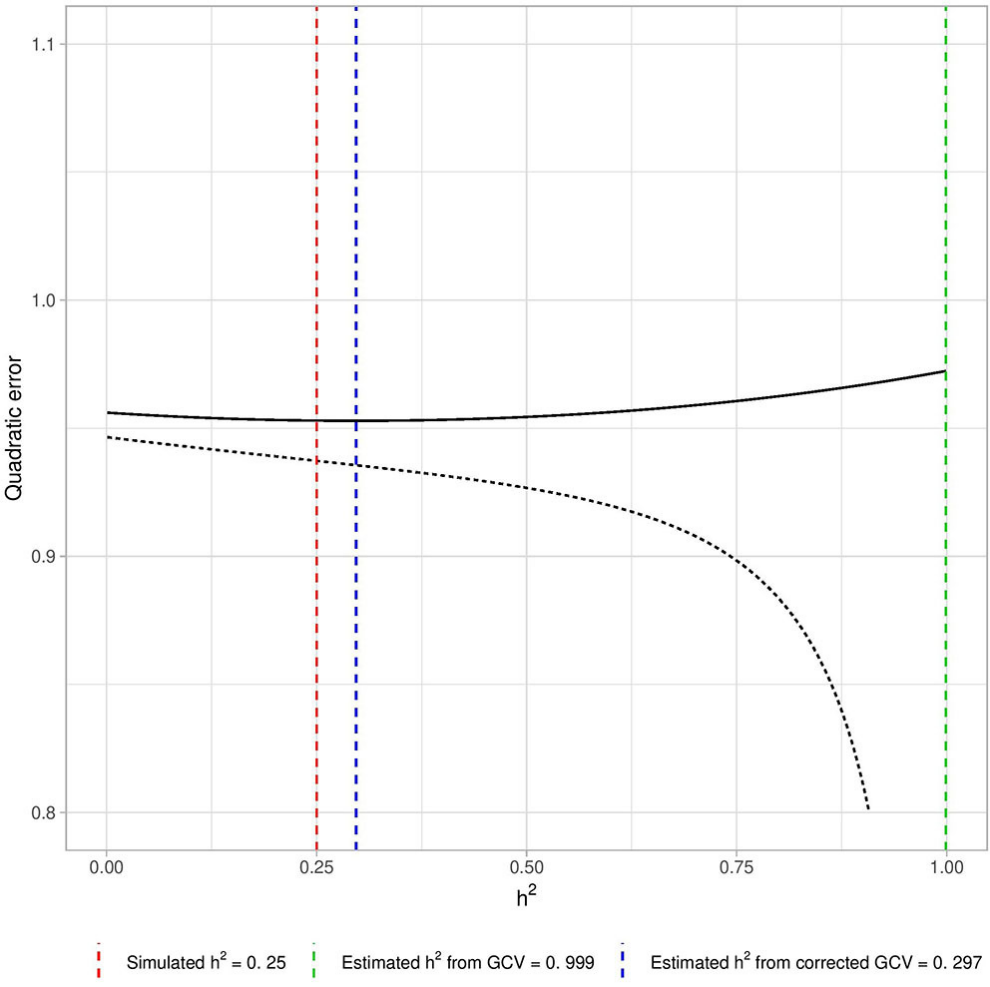
Example of biased estimation by GCV if *p* > *n*. We computed the GCV error curve with *n* = 1, 000 individuals, *p* = 10, 000 causal variants and simulated heritability $h_{sim}^{2}=0.25$. We used a grid of λ corresponding to the grid of heritability {0.01, 0.02, …, 0.99} using the link described in Equation (6) and computed the GCV error for those λ after empirical standardization of the data (dotted line). The λ that minimizes the GCV error corresponds to the heritability estimation. Here the GCV error tends to its minimum as *h*^2^ tends to 1, and heritability is thus largely over-estimated. The plain line corresponds to the GCV error obtained after correction of this bias by the projection approach (see section 2.1.3), which provides a satisfactory estimation of *h*^2^. The three vertical lines correspond respectively to the simulated heritability (red line), the heritability estimated using uncorrected GCV (green line) and the heritability estimated using corrected GCV (blue line).

Figure 1 shows the GCV error (dotted line). Heritability is strongly overestimated. The GCV error appears to tend toward its minimum as λ approaches 0 (i.e., when *h*^2^ tends to 1).

This is a direct consequence of the empirical standardization of **M** and of the phenotype. By centering the columns of **M** with the empirical means of those columns, a dependency is introduced, and each line of the resulting standardized genotype matrix **Z** becomes a linear combination of all the others. The same phenomenon of dependency can be observed with the phenotype when using empirical standardization. Given the nature of the LOO in general (where each individual is considered successively as a validation set), this kind of standardization introduces a link between the validation set and the training set at each step: the “validation set individual” can be written as a linear combination of the individuals in the training set. In high dimension, this dependency leads to ${\text{err}}^{LOO}\overset{\text{​}}{\underset{\lambda \to 0}{\to }}0$ (see section 4 in Supplementary Material), due to over-fitting occurring in the training set.

From a GCV perspective, a related consequence of the empirical centering of the genotype data is that the matrix **ZZ**^*T*^ has at least one null eigenvalue and an associated constant eigenvector in a high dimensional setting (see section 4 in Supplementary Material). This has a direct impact on GCV: using the singular value decomposition of the empirically standardized matrix **Z** = **UDV**^*T*^ with $\text{U}\in O_{n}\left(\mathbb{R}\right)$, $\text{V}\in O_{p}\left(\mathbb{R}\right)$ two orthogonal squared matrices spanning, respectively, the lines and columns spaces of **Z** while $\text{D}\in M_{n,p}\left(\mathbb{R}\right)$ is a rectangular matrix with singular values {*d*_1_, …, *d*_*n*_} on the diagonal. In a high dimensional context: ${\text{err}}^{GCV}\left(\text{y},\text{Z},\lambda \right)\overset{d_{n}^{2}=0}{\underset{\lambda \to 0}{\to }}{\left({{𝟙}_{n}}^{T}\text{y}\right)}^{2}$. Performing the “naive” empirical centering of the phenotype results in

$$\begin{array}{ll} {\text{err}}^{GCV}\left(\text{y}-\hat{\mu }{𝟙}_{n},\text{Z},\lambda \right) & \overset{d_{n}^{2}=0}{\underset{\lambda \to 0}{\to }}{\left({{𝟙}_{n}}^{T}\text{y}-{{𝟙}_{n}}^{T}\hat{\mu }{𝟙}_{n}\right)}^{2}=0. \end{array}$$

The very same problem is observed for a more general model with covariates (see section 4 in Supplementary Material).

#### 2.1.3. A First Solution Using Projection

A better solution for dealing with the intercept [and a matrix of covariates $\text{X}\in M_{n,r}\left(\mathbb{R}\right)$] in ridge regression is to use a projection matrix as a contrast and to work on the orthogonal of the space spanned by the intercept (and the covariates).

Contrast matrices are a commonly used approach in the field of mixed models for REstricted Maximum Likelihood computations (REML) (Patterson and Thompson, 1971). REML provides maximum likelihood estimation once fixed effects are taken into account. Contrast matrices are used to “remove” fixed effects from the likelihood formula. If we are only interested in the estimation of the component of variance, we do not even need to make this contrast matrix explicit: any semi-orthogonal matrix $\text{C}\in M_{n-r-1,n}\left(\mathbb{R}\right)$ such that $\text{C}{\text{C}}^{T}={\text{I}}_{n-r-1}$ and **C** × (μ***1*_*n*_**+**X**β) = 0_*n*−*r*−1_ provides a solution (see section 6 in Supplementary Material for details). In a ridge regression context, an explicit expression of $\hat{u}$ is needed for choosing the optimal complexity. An explicit form for **C** is therefore necessary.

In the presence of covariates, a QR decomposition can be used to obtain an explicit form for **C** (see section 5 in Supplementary Material for details). In the special case of an intercept without covariates, there is a convenient choice of **C**. Since the eigenvector of **ZZ**^*T*^ associated with the final null eigenvalue is constant, $\text{C}={\left[{\text{U}}_{1},...,{\text{U}}_{n-1}\right]}^{T}\in M_{n-1,n}\left(\mathbb{R}\right)$ is a contrast matrix adapted for our problem. Additionally, by considering **CZ** instead of **Z**, we have $\text{C}\text{Z}={\text{D}}_{-n}{\text{V}}^{T}\to \text{C}\text{Z}{\text{Z}}^{T}{\text{C}}^{T}={\text{D}}_{-n}{\text{D}}_{-n}^{T}$ with **D**_−*n*_ the matrix **D** deprived of row *n*. This choice of contrast matrix thus simplifies the GCV formula and allows extremely fast computation.

#### 2.1.4. A Second Solution Using 2 Data Sets

Dependency between individuals can be a problem when we use the same data for the standardization (including the estimation of potential covariate effects) and for the estimation of the genetic effects. This can be overcome by partitioning our data. Splitting our data into a standardization set and a training set, we will first use the standardization set to estimate the mean and the standard deviation of each variant, the intercept, and the potential covariate effects. Those estimators will then be used to standardize the training set on which GCV can then be applied.

This method has two main drawbacks. The first is that the estimation of the non-penalized effects is done independently of the estimation of the genetic effects, even though in practice we do not expect covariates to be highly correlated with variants. The other drawback is that it reduces the number of individuals for the heritability estimation (which is very sensitive to the number of individuals). This approach therefore requires a larger sample than when using projection.

### 2.2. Prediction vs. Heritability in the Context of Small Additive Effects

Ridge regression helps to highlight the link between heritability and prediction accuracy. What is the relation between the two concepts? Is prediction accuracy an increasing function of heritability?

In a machine learning setting, we have training and testing sets. The classical bias-variance trade-off formulation considers the expectation of the loss over both the training set and the test individual phenotype. It breaks down the prediction error into three terms commonly called variance, bias, and irreducible error. In this paper we do consider the genotypes of the training set as fixed and the genotype of a test individual as random, and somewhat abusively continue to employ the terms variance, bias, and irreducible error:

$$\begin{array}{l} {𝔼}_{{\text{y}}_{tr},y_{te},z_{te}}\left[{\left(y_{te}-{\hat{y}}_{te}\right)}^{2}\right]={𝔼}_{z_{te}}\left[{𝔼}_{{\text{y}}_{tr},y_{te}|z_{te}}\left[{\left(y_{te}-{\hat{y}}_{te}\right)}^{2}\right]\right]\\ \;={𝔼}_{z_{te}}\left[\text{var}\left(y_{te}|z_{te}\right)+\text{var}\left({\hat{y}}_{te}|z_{te}\right)\right]+\\ \;{𝔼}_{z_{te}}\left[{\left({𝔼}_{{\text{y}}_{tr}|z_{te}}\left[{\hat{y}}_{te}\right]-{𝔼}_{y_{te}|z_{te}}\left[y_{te}\right]\right)}^{2}\right]. \end{array}$$

where the index _*tr*_ refers to the training set, while _*te*_ refers to the test set.

Assuming a training set genotype matrix $\text{Z}\in M_{n,p}\left(\mathbb{R}\right)$ (without index _*tr*_ to lighten notations) whose columns have zero mean and unit variances, we denote ${\text{K}}_{\lambda }={\left({\text{Z}}^{T}\text{Z}+\lambda {\text{I}}_{p}\right)}^{-1}{\text{Z}}^{T}$. Assuming the independence of the variants 𝔼_*z*_*te*__[*z*_*te*_] = 0_*p*_ and var(*z*_*te*_) = **I**_*p*_, irreducible error, variance, and bias become:

$$\begin{array}{l} \;{𝔼}_{z_{te}}\left[\text{var}\left(y_{te}|z_{te}\right)\right]=\sigma^{2}\\ \;{𝔼}_{z_{te}}\left[\text{var}\left({\hat{y}}_{te}|z_{te}\right)\right]=\sigma^{2}\text{tr}\left({\text{K}}_{\lambda }{\text{K}}_{\lambda }^{T}\right)\\ {𝔼}_{z_{te}}\left[{\left({𝔼}_{{\text{y}}_{tr}|z_{te}}\left[{\hat{y}}_{te}\right]-{𝔼}_{y_{te}|z_{te}}\left[y_{te}\right]\right)}^{2}\right]=u^{T}{\left({\text{K}}_{\lambda }\text{Z}-{\text{I}}_{p}\right)}^{2}u. \end{array}$$

where *u* is the vector of the ridge parameters.

Since individuals are assumed to be unrelated, the covariance matrix of the individuals is diagonal. The covariance matrix of the variants is also diagonal, since variants are assumed independent. Assuming scaled data, **ZZ**^*T*^ and **Z**^*T*^**Z** are the empirical estimations of covariance matrices of respectively the individuals and the variants (up to a *p* or *n* scaling factor). Two separate situations can be distinguished according to the *n*/*p* ratio. In the high-dimensional case where *p* > *n*, the matrix **ZZ**^*T*^ estimates well the individuals' covariance matrix up to a factor *p*. Where *n* > *p*, on the other hand, **Z**^*T*^**Z** estimates well the covariance matrix of variants up to a factor *n*. Eventually, $\text{Z}{\text{Z}}^{T}≃p{\text{I}}_{n}$ when *n* < *p* and ${\text{Z}}^{T}\text{Z}≃n{\text{I}}_{p}$ when *n* > *p*.

Assuming further that

- ∀*i* ∈ [[1, *n*]] var(*y*_*i*_) = 1, we then have σ^2^ = 1 − *h*^2^,
- heritability is equally distributed among normalized variants i.e., $\forall j\in 〚1,p〛\;\text{var}\left(u_{j}\right)=\frac{h^{2}}{p}$ (which is indeed the mixed model hypothesis),
- $u^{T}u≃p\times \frac{h^{2}}{p}$ and (**Z***u*)^*T*^(**Z***u*) ≃ *nh*^2^,

the expected prediction error can be stated more simply, according to the $\frac{n}{p}$ ratio (see section 8 in Supplementary Material for details):

(11) $$\begin{array}{l} {𝔼}_{{\text{y}}_{tr},y_{te},z_{te}}\left[{\left(y_{te}-{\hat{y}}_{te}\right)}^{2}\right]≃\{\begin{array}{c} 1-\frac{n}{p}{\left(h^{2}\right)}^{2},\text{if }p\geq n\;\\ \left(1-h^{2}\right)\frac{1+\frac{n}{p}h^{2}}{1+h^{2}\left(\frac{n}{p}-1\right)},\text{ otherwise}. \end{array} \end{array}$$

**Figure 2:**
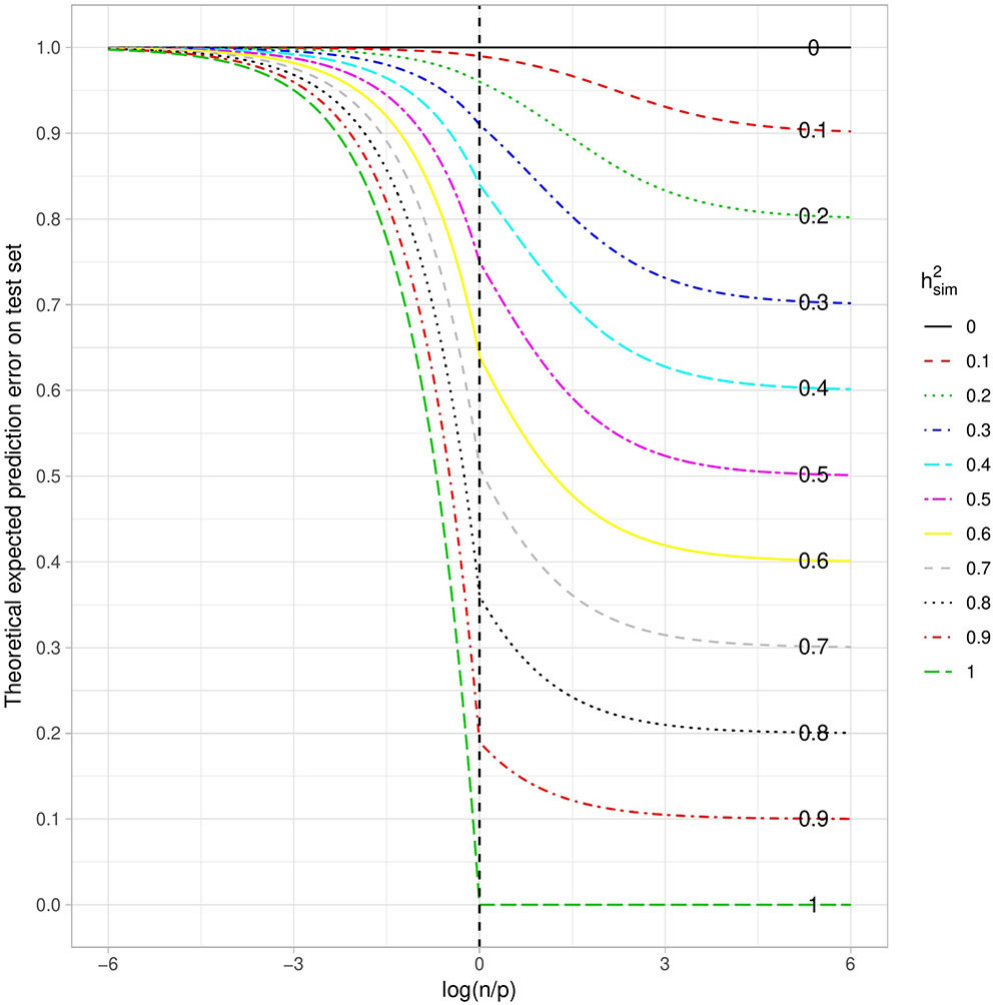
Theoretical Quadratic Error with respect to the log ratio of the number of individuals over the number of variants in the training set. Each curve corresponds to a given heritability (in the narrow sense). Note that the total variance is assumed to be 1.

When considering the theoretical quadratic error with respect to the log ratio of the number of individuals over the number of variants in the training set (Figure 2), as expected we have a decreasing function. This means that the larger the number of individuals in the training sample, the smaller the error. We also observe that the higher the heritability, the smaller the error. Both of these things are intuitive, and as a consequence the error tends toward the irreducible error when *n* becomes much larger than *p*. What is more surprising is that the prediction error is close to the maximum, whatever the heritability, when *n* is much smaller than *p*. Paradoxically, even with the highest possible heritability, if the number of variants is too large in relation to the number of individuals, no prediction is possible. This can be explained by the fact that the penalization plays a very important role in that case and thus strongly increases the bias, while reducing the variance. The squared bias and the variance with respect to the log ratio of the number of individuals over the number of variants in the training set are shown in Supplementary Figures 3, 4. The irreducible error is only a function of heritability and is not affected by the dimension of the training set.

Similarly, the prediction error can be computed on the training set instead of on the test set. Using the same assumptions as before, the expected prediction error on the training set can be approximated by:

$$\begin{array}{l} {𝔼}_{{\text{y}}_{tr}}\left[\frac{1}{n}{\left({\text{y}}_{tr}-{\hat{\text{y}}}_{tr}\right)}^{T}\left({\text{y}}_{tr}-{\hat{\text{y}}}_{tr}\right)\right]\\ ≃\{\begin{array}{c} {\left(1-h^{2}\right)}^{2}\text{if}p>n,\\ 1-2\frac{n}{n+\lambda }\left(\frac{p}{n}\left(1-h^{2}\right)+h^{2}\right)+{\left(\frac{n}{n+\lambda }\right)}^{2}\left(\frac{p}{n}\left(1-h^{2}\right)+h^{2}\right)\text{otherwise.} \end{array} \end{array}$$

A graph similar to Figure 2 for this expected error can be found in Supplementary Figure 5. Interestingly, when *p* > *n*, the error on the training set does not depend on the *n*/*p* ratio. When *n* becomes greater than *p*, it increases and tends toward the irreducible error 1 − *h*^2^ when *n* ≫ *p*. As shown in Figure 2, the error on the test set is always higher than the irreducible error and thus higher than the error on the training set, which is a sign of over-fitting. However, the difference between the error on the test set and the error on the training set is a decreasing function of the *n*/*p* ratio, which is linear when *p* > *n* and tends toward zero when *n* ≫ *p*.

Another popular way of looking at the predictive accuracy is to consider the squared correlation between *y*_*te*_ and $\hat{y}$_*te*_ (Goddard, 2009; Daetwyler et al., 2010):

$$\begin{array}{l} {\text{corr}}^{2}\left(y_{te},{\hat{y}}_{te}\right)=\frac{{\text{cov}}^{2}\left(y_{te},{\hat{y}}_{te}\right)}{\text{var}\left[y_{te}\right]\text{var}\left[{\hat{y}}_{te}\right]}. \end{array}$$

Although correlation and prediction error both provide information about the prediction accuracy, correlation may have an interpretation that is intuitive, but it does not take the scale of the prediction into account. From a predictive point of view, this is clearly a disadvantage. Considering *y*_*te*_, *z*_*te*_, and *y*_*tr*_ to be random, and using the same assumptions that were made in relation to prediction error, the three terms of the squared correlation become:

$$\begin{array}{l} {\text{cov}}^{2}\left(y_{te},{\hat{y}}_{te}\right)={\left(u^{T}{\text{K}}_{\lambda }{\text{Z}}_{tr}u\right)}^{2},\\ \text{ var}\left[{\hat{y}}_{te}\right]=\text{tr}\left({\text{K}}_{\lambda }^{T}{\text{K}}_{\lambda }\times \sigma^{2}{\text{I}}_{n}\right)+{\left({\text{Z}}_{tr}u\right)}^{T}{\text{K}}_{\lambda }^{T}{\text{K}}_{\lambda }\left({\text{Z}}_{tr}u\right),\\ \text{ var}\left[y_{te}\right]=1. \end{array}$$

Like in the case of prediction error, replacing **ZZ**^*T*^ or **Z**^*T*^**Z** by their expectations, the squared correlation simplifies to:

(12) $$\begin{array}{l} {\text{corr}}^{2}\left(y_{te},{\hat{y}}_{te}\right)≃\{\begin{array}{c} \frac{n}{p}{\left(h^{2}\right)}^{2}\text{ if }n<p,\\ \frac{{\left(h^{2}\right)}^{2}}{\frac{p}{n}\left(1-h^{2}\right)+h^{2}}\text{ otherwise}. \end{array} \end{array}$$

**Figure 3:**
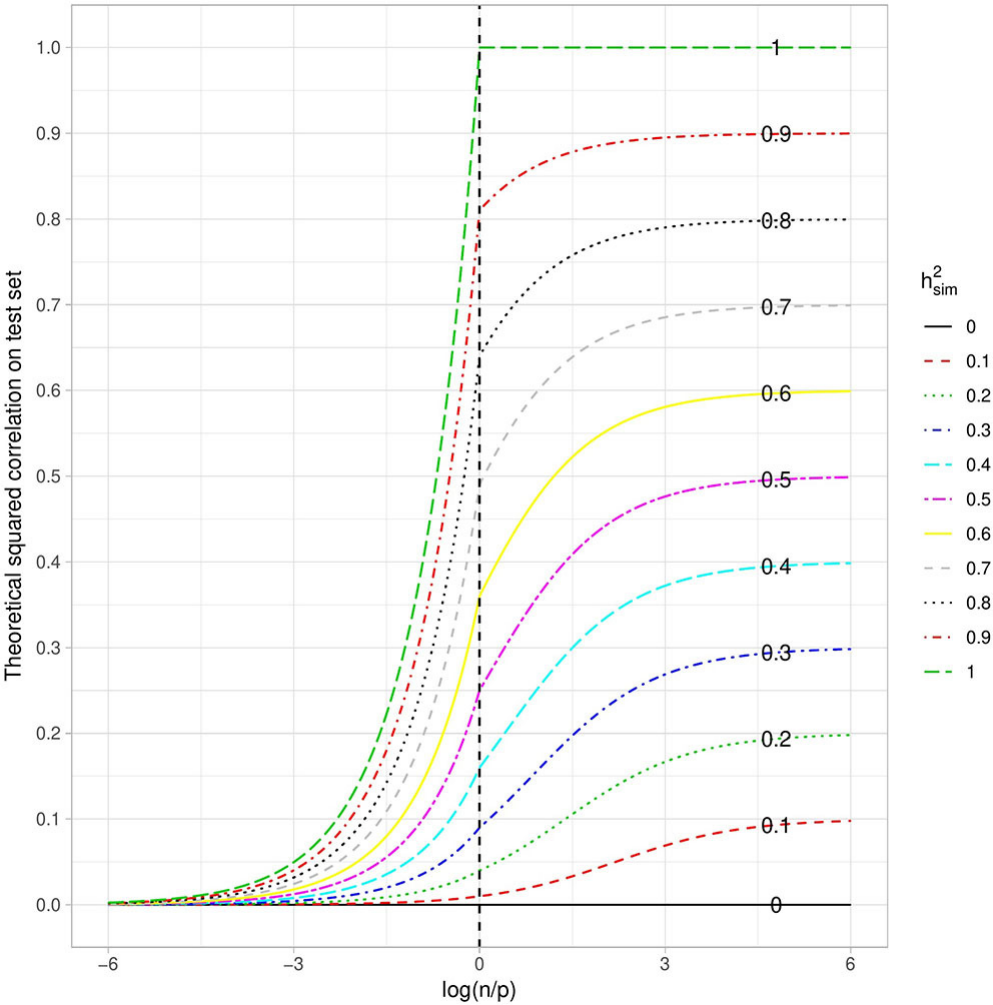
Theoretical squared correlation between phenotype and its prediction with respect to the log ratio of the number of individuals over the number of variants in the training set. Each curve corresponds to a given heritability (in the narrow sense).

When considering this theoretical squared correlation with respect to the log ratio of the number of individuals over the number of variants in the training set (Figure 3), we have, as expected, an increasing function. Similarly, the higher the heritability, the higher the squared correlation. We also observe that when *n* ≫ *p*, the squared correlation tends toward the simulated heritability. Conversely, when *p* ≫ *n*, it is close to zero whatever the heritability.

### 2.3. Simulations and Real Data

Since narrow-sense heritability is a quantity that relates to a model, we will first illustrate our contributions via simulations where the true model is known. We perform two different types of simulation: fully synthetic simulations where both genotypes and phenotypes are drawn from statistical distributions, and semi-synthetic simulations where UK Biobank genotypes are used to simulate phenotypes. We also illustrate our contributions using height and body mass index (BMI) from the UK Biobank dataset.

We first assess the performance of GCV for heritability estimation and then look at the accuracy of the prediction when the ratio of the number of individuals to the number of variants varies in the training set.

#### 2.3.1. UK Biobank Dataset

The present analyses were conducted under UK Biobank data application number 45,408. The UK Biobank dataset consists of ≃ 784 K autosomal SNPs describing ≃ 488 K individuals. We applied relatively stringent quality control and minor allele frequency filters to the dataset (callrate for individuals and variants > 0.99; *p*-values of Hardy-Weinberg equilibrium test > 1e-7; Minor Allele Frequency > 0.01), leading to 473,054 and 417,106 remaining individuals and SNPs, respectively.

Two phenotypes were considered in our analyses: height (standing) and BMI. In order to have a homogeneous population for the analysis of these real phenotypes, we retained only those individuals who had reported their ethnicity as white British and whose Principal Component Analysis (PCA) results obtained by UK Biobank were consistent with their self-declared ethnicity. In addition, each time we subsampled individuals we removed related individuals [one individual in all pairs with a Genetic Relatedness Matrix (GRM) coefficient >0.025 was removed], as in Yang et al. (2011b) in order to avoid confusion between shared genetic factors and shared environmental factors. Several covariates were also considered in the analysis of these phenotypes: the sex, the year of birth, the recruitment center, the genotyping array, and the first 10 principal components computed by UK Biobank.

#### 2.3.2. Synthetic Genotype Data

The synthetic genotype matrices are simulated as in Golan et al. (2014) and de Vlaming and Groenen (2015). This corresponds to a scenario with independent loci or perfect linkage equilibrium. To simulate synthetic genotypes for *p* variants, we first set a vector of variant frequencies *f* ∈ ℝ^*p*^, with these frequencies independently following a uniform distribution U([0.05,0.5]). Individual genotypes are then drawn from binomial distributions with proportions *f*, to form the genotype matrix **M**. A matrix of standardized genotypes Z^*^ can be obtained by standardizing **M** with the true variant frequencies *f*.

#### 2.3.3. Simulations to Assess Heritability Estimation Using GCV

We consider both synthetic and real genetic data, and simulate associated phenotypes.

In the two simulation scenarios we investigate the influence on heritability estimation of the following three parameters: the shape of the genotype matrix in the training set (the ratio between *n* the number of individuals and *p* the number of variants), the fraction of variants with causal effects *f*_*c*_, and the true heritability $h_{sim}^{2}$. The tested levels of these quantities are shown in Table 1.

**Table 1:** Table of the parameters sets of the simulations.

**Parameters**	**Levels**
n/p	Simulation: 1,000/10,000; 5,000/10,0000; 10,000/500,000
	Data-based: 1,000/10,000; 5,000/10,0000; 10,000/417,106
*f*_*c*_	0.1; 0.5; 1
$h_{sim}^{2}$	{0.1, …, 0.9}

*n/p: the ratio of the dimensions of the genotype matrix. f_c_: proportion of causal variants. $h_{sim}^{2}$: simulated heritability*.

For each simulation scenario and for a given a set of parameters (*n*,*p*,*f*_*c*_,$h_{sim}^{2}$), the simulation of the phenotype starts with a matrix of standardized genotypes (either a synthetic genotype matrix Z^*^ standardized with the true allele frequencies, as described in section 2.3.2, or a matrix of empirically standardized genotypes **Z** obtained from UK Biobank data). To create the vector of genotype effects *u*, *p* × *f*_*c*_ causal SNPs are randomly sampled and their effects are sampled from a multivariate normal distribution with zero mean and a covariance matrix τ**I**_*p*×_*f*__*c*__ (where $\tau =\frac{h_{sim}^{2}}{p\times f_{c}}$), while the remaining *p*×(1 − *f*_*c*_) effects are set to 0. The vector of environmental effects **e** is sampled from a multivariate normal distribution with zero mean and a covariance matrix $\sigma^{2}{\text{I}}_{n}$, where $\sigma^{2}=1-h_{sim}^{2}$. The phenotypes are then generated as **y** = Z^*^u + **e** and **y** = **Z***u* + **e**, for the fully synthetic scenario and the semi-synthetic scenario, respectively. A standardization set of 1,000 individuals (that will be used for the GCV approach based on two datasets) is also generated for each scenario in the same way.

Applying GCV to large-scale matrices can be extremely time-consuming, since it requires the computation of the GRM associated with Z^*^ or **Z** and the eigen decomposition of the GRM. For this reason we employed the same strategy as de Vlaming and Groenen (2015) in order to speed up both simulations and analyses by making it possible to test more than one combination of simulation parameters. We simulated an (*n*_*max*_ = 10,000 × *p*_*max*_ = 500,000) genotype matrix for the training set in the fully synthetic scenario and used this simulated matrix for all the 9 × 3 × 3 = 81 ($h_{sim}^{2}\times f_{c}\times n/p$) parameter combinations. Similarly, we sampled *n*_*max*_ = 10,000 individuals from the UK Biobank dataset to obtain an (*n*_*max*_ = 10,000 × *p*_*max*_ = 417,106) genotype matrix for the training set in the semi-synthetic scenario. Smaller matrices were then created from a subset of these two large matrices (note that for subsets of the real genotype matrix we took variants in the original order to keep the linkage disequilibrium structure). Consequently, computation of the GRM and its eigen decomposition needed to be performed only once for each *n*/*p* ratio considered. The fully synthetic and the semi-synthetic scenarios were each replicated 30 times.

#### 2.3.4. Simulations to Assess Prediction Accuracy

We performed fully synthetic simulations for different ratios $\frac{n}{p}$ in order to study the behavior of the mean prediction error and the correlation between the phenotype and its prediction. We considered a training set of size *n* = 1,000, and a test set of size *n*_*te*_ = 5,000. The maximum number of variants was set to *p*_*max*_ = 50,000 and the heritability to *h*^2^ = 0.6. We first simulated a global allelic frequency vector $f\sim U_{p_{max}}\left(0.05,0.5\right)$ and a global vector of genetic effects $u\sim N\left(p_{max},\frac{h^{2}}{p_{max}}{\text{I}}_{p_{max}}\right).$

For each subset of variants of size *p* < *p*_*max*_, we selected a vector of genetic effects composed of the *p* first components of *u* multiplied by a $\sqrt{\frac{p_{max}}{p}}$ factor assuring a total variance of 1 and $\text{var}\left(u^{p}\right)=\frac{h^{2}}{p}{\text{I}}_{p}$: $u^{p}=\left(u_{1},...,u_{p}\right)\times \sqrt{\frac{p_{max}}{p}}$. The genotype matrix **M**_*te*_ was then simulated and its normalized version ${\text{Z}}_{te}^{*}$ computed as described in section 2.3.2. The normalization used the first *p* components of *f*. The noise vector ${\text{e}}_{te}\sim N\left({\text{0}}_{n_{te}},\left(1-h^{2}\right){\text{I}}_{n_{te}}\right)$ and a vector of phenotypes ${\text{y}}_{te}={\text{Z}}_{te}^{*}u^{p}+{\text{e}}_{te}$ were eventually simulated.

We generated 300 training sets by simulating the normalized genotype matrix, noise, and phenotype using the same process as for the test set. Here, the training set index is denoted as *k*. A prediction ${\hat{\text{y}}}_{te,k}$ for the test set was made with each training set using the ridge estimator of **u**^*p*^ obtained with $\lambda =p\frac{1-h^{2}}{h^{2}}$, and the following empirical quantities were estimated: ${\text{err}}_{p}=\frac{1}{300}\underset{k}{\sum }\frac{1}{n_{te}}\|{\text{y}}_{te,k}-{\hat{\text{g}}}_{p}{\|}_{2}^{2}$, ${\text{bias}}_{p}^{2}=$ $\frac{1}{n_{te}}\underset{i\in 〚1,n_{te}〛}{\sum }{\left({\left[{\text{Z}}_{te}u^{p}-{\hat{\text{g}}}_{p}\right]}_{i}\right)}^{2}$ and ${\text{var}}_{p}=\frac{1}{300}\underset{k}{\sum }\frac{1}{n_{te}}$$\|{\hat{\text{y}}}_{te,k}-{\hat{\text{g}}}_{p}{\|}_{2}^{2}$, where ${\hat{\text{g}}}_{p}=(\frac{1}{300}\sum_{k\in 〚1,300〛}$ ${{\left[{\hat{\text{y}}}_{te,k}\right]}_{i})}_{i\in 〚1,n_{te}〛}$. The squared correlation between ${\hat{\text{y}}}_{te,k}$ and **y**_*te, k*_ was also estimated.

We considered the following numbers of variants:

$$\begin{array}{l} p\in \{50,000;25,000;16,667;12,500;10,000;5,000;3,333;\\ \;2,500;2,000;1,667;1,429;1,250;1,111;1,000;500;\\ \;136;79;56;43;35;29;25;22;20\}. \end{array}$$

### 2.4. Prediction of Height and BMI Using UK Biobank Data

To experiment on UK Biobank for assessing the prediction accuracy, for each phenotype we considered three sets of data: a training set for the purpose of learning genetic effects, a standardization set for learning non-penalized effects (covariates and intercept), and a test set for assessing predictive power. Pre-treatment filters (as described in section 2.3.1) were systematically applied on the training set. We computed the estimation of genetic effects using the projection-based approach to take into account non-penalized effects, where the penalty parameter was obtained by GCV with the same projection approach:

$$\begin{array}{l} {\hat{u}}_{R}={\text{Z}}_{tr}^{T}{\text{C}}_{tr}^{T}{\left({\text{C}}_{tr}{\text{Z}}_{tr}{\text{Z}}_{tr}^{T}{\text{C}}_{tr}^{T}+{\hat{\lambda }}_{GCV}{\text{I}}_{n-r}\right)}^{-1}{\text{C}}_{tr}{\text{y}}_{tr}. \end{array}$$

We then estimated non-penalized effects (here **X** contains the intercept):

(13) $$\begin{array}{l} \hat{\beta }={\left({\text{X}}_{std}^{T}{\text{X}}_{std}\right)}^{-1}{\text{X}}_{std}^{T}\left({\text{y}}_{std}\right). \end{array}$$

Finally, we applied these estimations on the test set:

$$\begin{array}{l} {\hat{\text{g}}}_{te}={\text{Z}}_{te}{\hat{u}}_{R},\\ \;{\hat{\text{f}}}_{te}={\text{X}}_{te}\hat{\beta },\\ {\tilde{\text{y}}}_{te}={\text{y}}_{te}-{\hat{\text{f}}}_{te}, \end{array}$$

in order to compute the Mean Square Error = $\frac{1}{n_{te}}{\left({\tilde{\text{y}}}_{te}-{\hat{\text{g}}}_{te}\right)}^{T}\left({\tilde{\text{y}}}_{te}-{\hat{\text{g}}}_{te}\right)$ between the phenotype residuals ${\tilde{\text{y}}}_{te}$ after removal of non-penalized effects and ${\hat{\text{g}}}_{te}$.

This procedure was performed for different ratios $\frac{n}{p}$ using different sized subsets of individuals for the training set, while keeping all the variants that passed pre-treatment filters (see Table 2).

**Table 2:** Size (number of individuals) of training, standardization, and test sets for assessing predictive power on real data.

**Set**	**Size**
Training	{1, 000;2, 000;5, 000;10, 000;20, 000}
Standardization	1,000
Test	1,000

For each number *n* of individuals considered in the training set, the sampling of these individuals was repeated several times, as seen in Table 3, in order to account for the variance of the estimated genetic effects due to sampling.

**Table 3:** Number of repetitions for the evaluation of the predictive power on real data.

Size of the training set	1,000	2,000	5,000	10,000	20,000
Number of repetitions	100	70	50	20	10

## 3. Results

### 3.1. Generalized Cross Validation for Heritability Estimation

#### 3.1.1. Simulation Results

**Figure 4:**
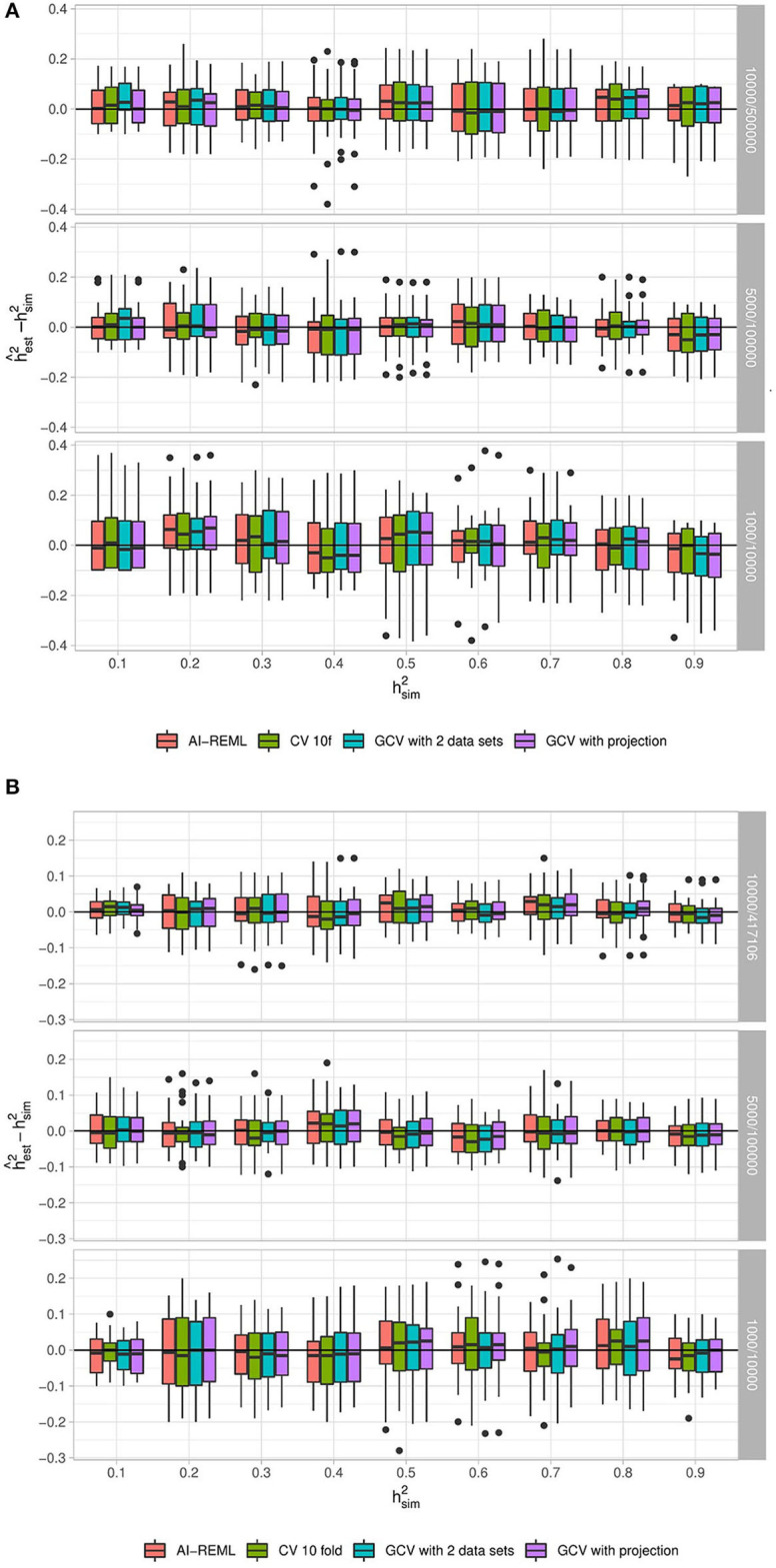
Distribution of $\left(h_{est}^{2}-h_{sim}^{2}\right)$ for different parameter combinations with 30 replications. **(A)** Corresponds to data simulated under the “fully synthetic” procedure, while **(B)** corresponds to the “semi-synthetic simulation” procedure. Each sub-panel corresponds to a different value of *n*/*p*. In both scenarios 10% of the variants have causal effects (i.e., *f*_*c*_ = 0.1). For each panel, the horizontal axis corresponds to the simulated heritability $h_{sim}^{2}\in \left\{0.1,...,0.9\right\}$ and the vertical axis corresponds to $\left(h_{est}^{2}-h_{sim}^{2}\right)$. Heritability estimations are done with the random effects model using AI-REML and with ridge regression using three approaches for the choice of λ: GCV with a projection correction, GCV with a two-dataset correction and a 10-fold cross-validation (CV 10f).

For the two simulation scenarios we look at the difference between the estimation of $h_{g}^{2}$ by GCV and the simulated heritability $h_{sim}^{2}$ in different configurations of study size *n*/*p*, $h_{sim}^{2}$ and the fraction of causal variants *f*_*c*_. Similarly, we look at the difference between the estimation by the classical mixed model approach and the simulated heritability. In our simulations *f*_*c*_ was seen to have no influence, and so only the influence of the remaining parameters is shown in Figure 4 and *f*_*c*_ is fixed at 10%. For full results, see Supplementary Figures 1, 2.

For the fully-simulated scenario, the two GCV approaches give very similar results and appear to provide an unbiased estimator of *h*^2^. They compare very well with the estimation of heritability by ridge regression with a 10-fold CV. Moreover, the variance of the GCV estimators does not appear higher than the variance of 10-fold CV.

In the case of the semi-synthetic simulations, here too both GCV approaches and the 10-fold CV provide a satisfactory heritability estimation. Our choice of using GCV in place of a classical CV approach for estimating heritability by ridge regression is therefore validated.

For both simulation scenarios we also note that the classical mixed model approach (using the AI-REML method in the gaston R package) gives heritability estimations that are very similar to those obtained using the GCV approaches. The value of simulated heritability does not appear to have a strong effect on the quality of the heritability estimation. On the other hand, the ratio *n*/*p* seems to have a real impact on estimation variance, with lower ratios leading to lower variances, which initially might appear surprising. One possible explanation for this is that in our simulations *n* increases as the ratio *n*/*p* decreases. Visscher and Goddard (2015) showed that the variance of the heritability is a decreasing function of *n*, which could explain the observed behavior.

#### 3.1.2. Illustration on UK Biobank

We now compare heritability estimations between the two GCV approaches and the classical mixed model approach for height and BMI, on a training set of 10,000 randomly sampled individuals (the training set being of the same size as for the simulated data). All three approaches take account of covariates and the intercept. The AI-REML approach also uses a projection matrix to deal with covariates. For the GCV approach based on two datasets, a standardization set of 1,000 individuals is also sampled, and for comparison purposes we have chosen to apply this two-set strategy to the classical mixed model approach as well.

Since the true heritability is of course unknown with real data, the sampling of the training and standardization sets is repeated 10 times in order to account for heritability estimation variability. Note that the SNP quality control and MAF filters were repeated at each training set sampling and applied to the standardization set.

**Figure 5:**
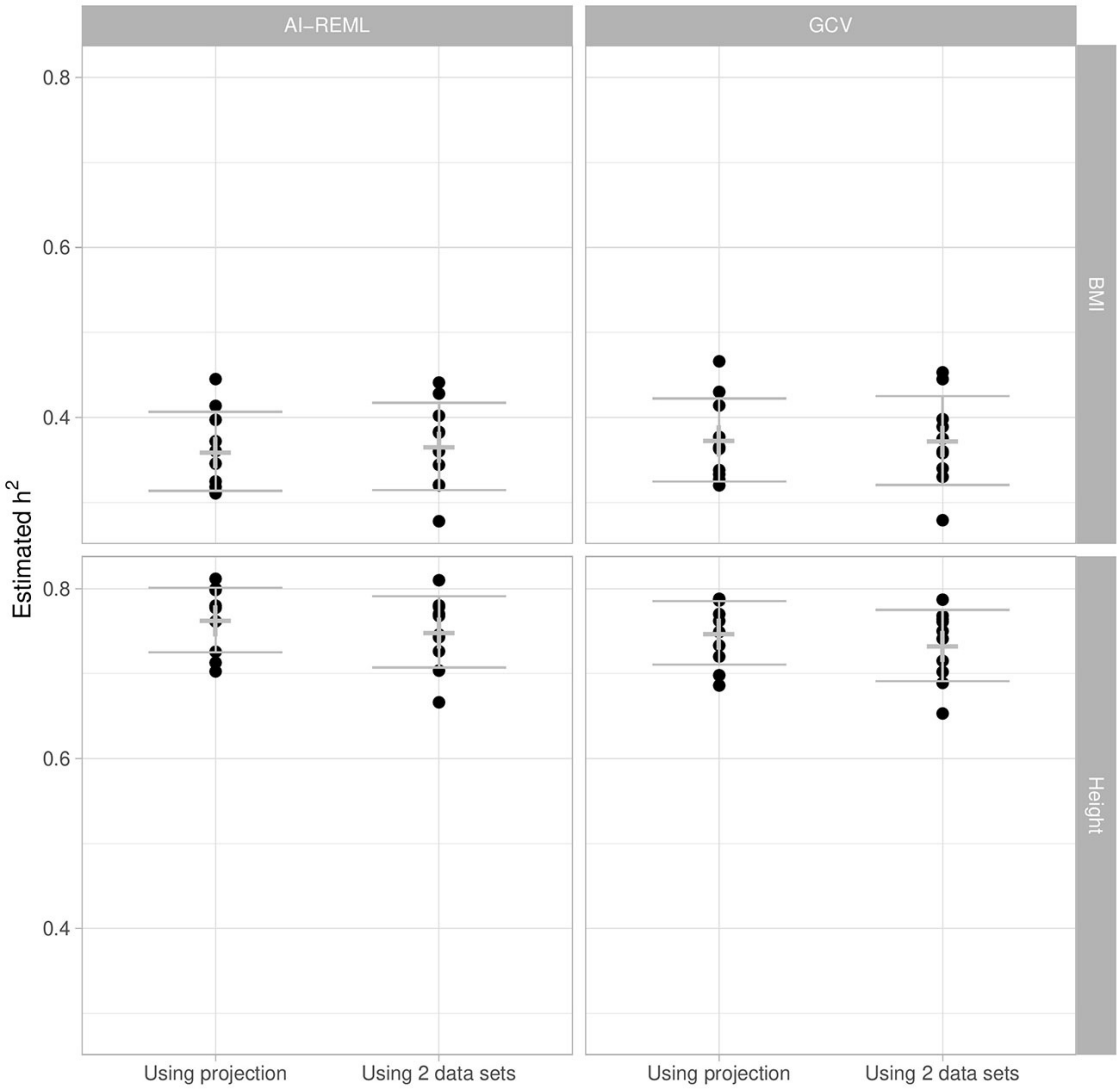
Heritability estimation of BMI and height using AI-REML and GCV, with the projection-based approach and with the two-set approach. We sub-sampled the original UK Biobank dataset 10 times for replication. The cross corresponds to the mean and the error bar to the mean ± one standard deviation.

Figure 5 shows that for each phenotype the two GCV approaches and the classical mixed model approach (AI-REML) give similar estimations. There is relatively little estimation variability, and any variability observed seems depend more on the individuals sampled for the training set than on the approach used.

### 3.2. Prediction vs. Heritability in the Context of Small Additive Effects

#### 3.2.1. Prediction From Synthetic Data

**Figure 6:**
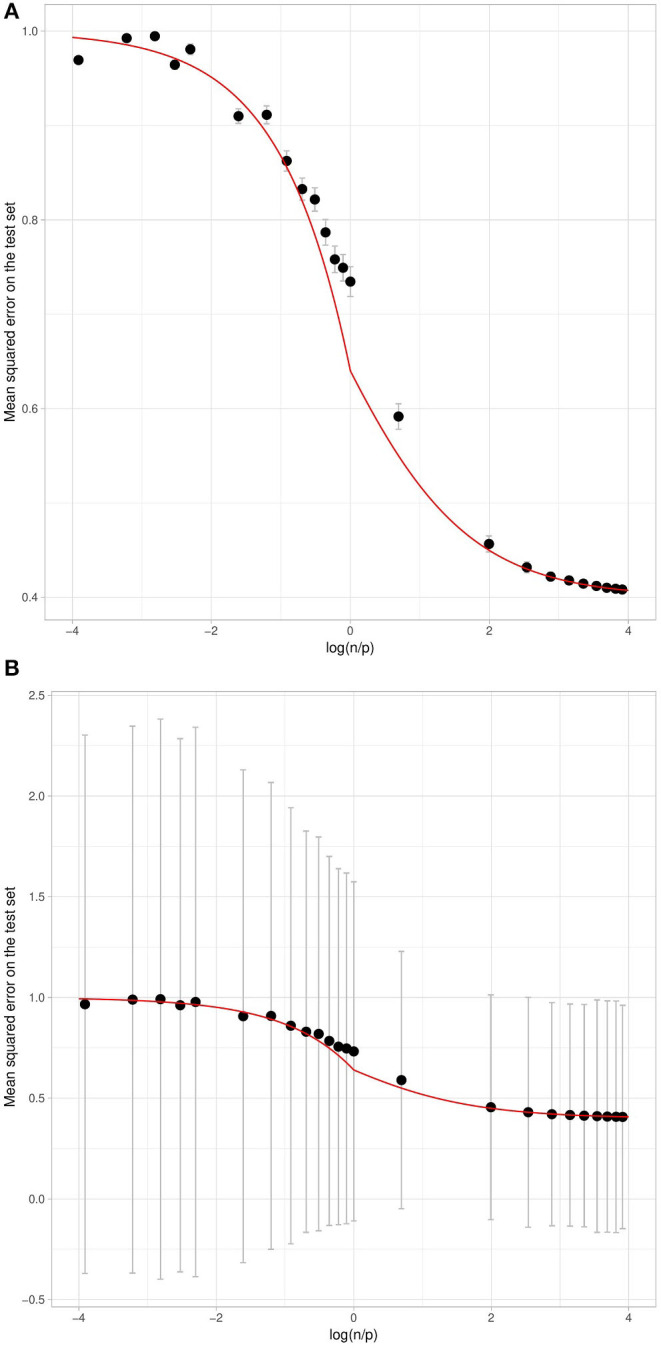
Mean Squared Error of the prediction on the test set with respect to $\text{log}\left(\frac{n}{p}\right)$, using simulated data with *h*^2^ = 0.6. The curves correspond to the theoretical link for *h*^2^ = 0.6. The black points correspond to the mean expectation for each value of $\text{log}\left(\frac{n}{p}\right)$ over 300 repetitions. The error bars in **(A,B)** correspond respectively to one standard deviation of the test set error obtained using two different aggregation strategies. On the left **(A)**, we consider an aggregation strategy where each of the 300 training sets results in a mean test set error, whereas on the right **(B)** each individual in the test set results in an error averaged over all training sets.

As expected, the mean of the test set error follows closely the theoretical curve when the $\log\frac{n}{p}$ varies (Figure 6). When *n* > *p*, the mean of the test set is close to the minimum possible error, which means that the ridge regression provides a reliable prediction on average.

Interestingly, if the mean error behaves as expected by our approximation, the standard deviation of the error may be very large. Figures 6A,B show the same mean error with different error bars. Figure 6A plots the error bars corresponding to the training set variation: the mean test set error is computed for each training set and the error bars show one standard deviation across the 300 training sets. Figure 6B plots the error bars corresponding to the variation of the errors across the test set.

The error bars in Figure 6B are much larger than those in Figure 6A, which shows that the variation in the prediction error is mostly due to the test individual whose phenotype we wish to predict, and depends little on the training set. This may be explained by the fact that the environmental residual term can be very large for some individuals. For these individuals the phenotype will be predicted with a very large error even when *n* ≫ *p*, that is to say when the genetic term is correctly estimated, irrespective of the training set.

**Figure 7:**
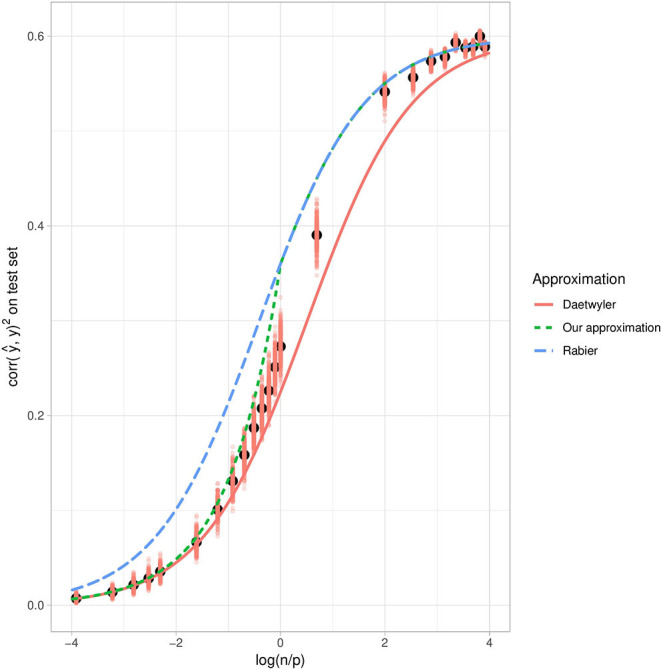
Mean squared correlation between the phenotype and its prediction on the test set with respect to $\text{log}\left(\frac{n}{p}\right)$, using simulated data with *h*^2^ = 0.6. The salmon points correspond to the evaluation of the squared correlation and the black points correspond to the mean expectation for each value of $\text{log}\left(\frac{n}{p}\right)$ over 300 repetitions. Red dots correspond to training set replications. The red plain curve corresponds to Daetwyler's approximation for *h*^2^ = 0.6, while the blue dashed curve corresponds to Rabier's approximation and the green dotted curve corresponds to ours.

The squared correlation between the phenotype and its prediction, as a function of $\text{log}\frac{n}{p}$, is also in line with our approximation (Figure 7). As expected, when *n* ≫ *p*, the squared correlation tends toward the simulated heritability. We compared our approximation with the approximation obtained by Daetwyler et al. (2008) and observed that although Daetwyler's approximation is very similar to ours when *p* ≫ *n*, our simulation results make Daetwyler's approximation appear under-optimistic when *n* ≫ *p*. Finally, we also compared our approximation with that obtained by Rabier et al. (2016), which is the same as ours when *n* > *p*. However, when *p* > *n*, Rabier's approximation appears over-optimistic.

#### 3.2.2. Prediction From UK Biobank Data

Let us consider the proposed theoretical approximation of the predictive power of ridge regression with respect to the *n*/*p* ratio applied to the UK Biobank data, for height and BMI residuals (after removal of covariate effects and intercept).

The two phenotypes differ considerably as regards heritability: we estimate by the projection-based GCV approach that 73.33% of height is “heritable” whereas only 33.91% of BMI is (on average over the 10 training samples of 20,000 individuals).

These estimated values are close to those currently found in the literature (Ge et al., 2017). It is important to note that the heritability estimation is strongly dependent on the filters. Variations of up to 20% were observed in the estimations when the filtering procedure setup was slightly modified.

A major difference between UK Biobank data and our simulations designed to check the proposed approximation lies in the strong linkage disequilibrium present in the human genome. Several papers have proposed using the effective number of independent markers to make adjustments in the multiple testing framework (Li et al., 2012), and we likewise propose adjusting our prediction model by taking into account an effective number of SNPs (*p*_*e*_). We estimate the effective $\frac{n}{p_{e}}$ ratio for each training set and for each considered *n* value using the observed mean square errors, the estimated heritability, and the theoretical relation in the case of independent variants ${𝔼}_{{\text{y}}_{tr},y_{te},z_{te}}\left[{\left(y_{te}-{\hat{y}}_{te}\right)}^{2}\right]=1-\frac{n}{p}{\left(h^{2}\right)}^{2}$ when *p* > *n*. We then use a simple linear regression to find the coefficient between these estimated $\frac{n}{p_{e}}$ ratios and the corresponding real $\frac{n}{p}$ ratios.

Table 4 shows different but close effective numbers of SNPs for the two phenotypes.

**Table 4:** Effective number of SNPs.

**Phenotype**	***p*/*p*_*e*_**
Height	5.01
BMI	3.48

**Figure 8:**
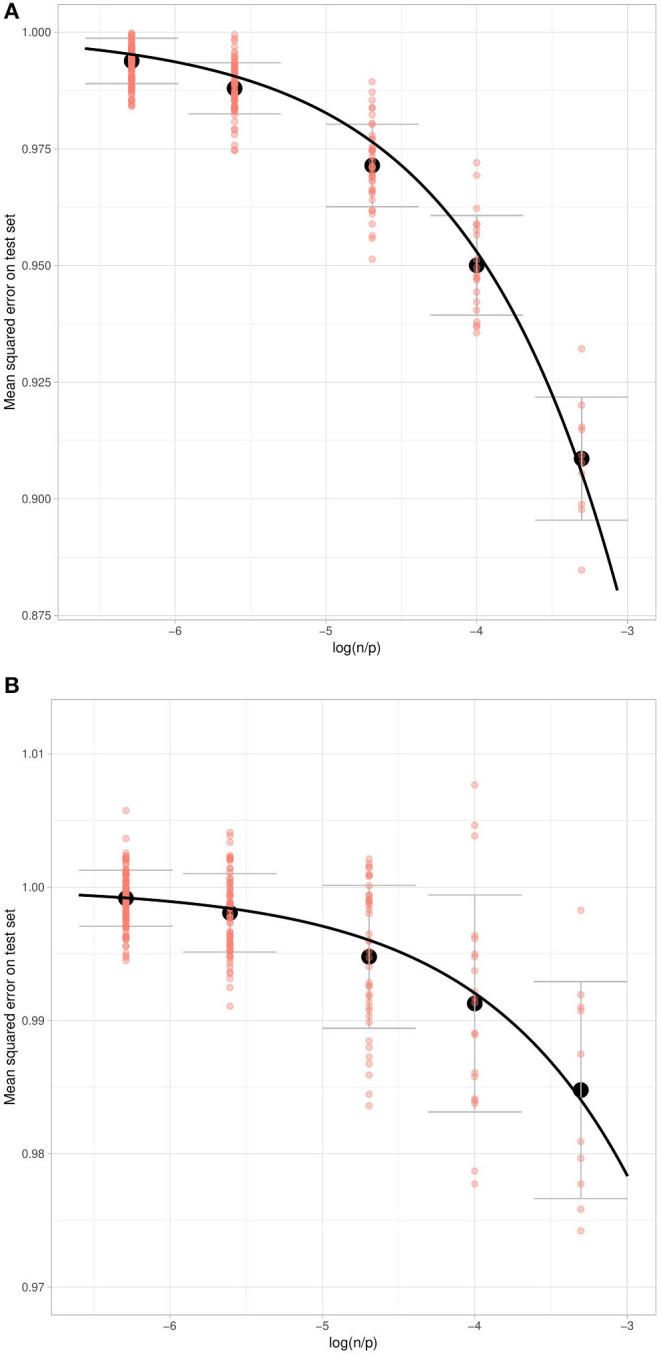
Normalized Mean Squared Error on the test set for the prediction of Height **(A)** and BMI **(B)** with respect to the log ratio of the number of individuals over the number of markers in the training set. Salmon dots correspond to training set replications, black dot to the mean of replications for different ratio and we show one standard deviation (over the training sets) of the mean test set error. The theoretical curves are fitted using the estimated heritability and an effective number of markers.

We also consider normalizing the test set errors using the mean square error of phenotype residuals (after removing non-penalized effects). Using this error normalization and adjusting the theoretical curve for an effective number of SNPs, we observe a close fit between the estimated errors on the test set and their theoretical values (Figure 8).

## 4. Discussion

In this work we investigated an alternative computation of genomic heritability based on ridge regression. We proposed a fast, reliable way to estimate the optimal penalization parameter of the ridge via Generalized Cross Validation adapted for high dimension. The genomic heritability estimated from the GCV gives results comparable to mixed model AIREML estimates. It clearly demonstrates that a predictive criterion allows a reliable choice of the penalization parameter and associated heritability, even when the prediction accuracy of ridge regression is low. Moreover, even though our approach does not formally consider Linkage Disequilibrium, simulations showed that it still provides reliable genomic heritability estimates in presence of realistic Linkage Disequilibrium.

We also provided theoretical approximations of the ridge regression prediction accuracy, in terms of both error and correlation between the phenotype and its prediction on new samples. These approximations perform well on synthetic data, in both high and low dimensions. They rely on the assumption that individuals and markers are independent in approximating the empirical covariance matrices. Our approximation of the prediction accuracy in terms of correlation proposes a good compromise between existing approximations. In particular, it exhibits similar performances to Daetwyler et al. (2008) when *p* > *n* and to Rabier et al. (2016) when *p* < *n*.

Our theoretical approximation of the prediction error is also consistent with the error observed on real genetic data when *p* > *n*, after adjusting for the effective number of independent markers. Unfortunately, due to computational issues, we were unable to perform the analysis in the *n* ≃ *p* case with real data. However, we observed that the prediction accuracy already reaches almost 15% of the heritability of height when *n*/*p* ≃ 5%, while De los Campos et al. (2013) suggested that its asymptotic upper bound is of the order of 20% of the heritability because of incomplete LD between causal loci and genotyped markers. Interestingly, ridge regression is not affected by correlated predictors, and consequently it is not affected by high LD between markers. When LD is high, this has the effect of reducing the degrees of freedom of the model (Dijkstra, 2014), which results in an improved prediction accuracy in comparison with a problem having the same number of independent predictors and the same heritability.

Although our approximations and simulation results tend to show that the prediction accuracy can reach the heritability value when *n* ≫ *p*, as already suggested by previous works (Daetwyler et al., 2008; de Vlaming and Groenen, 2015; Rabier et al., 2016), the large standard deviation of the prediction error that we observed between simulated individuals suggests that disease risk prediction from genetic data alone is not accurate at the individual level, even for a relatively high heritability value in the context of a small additive effect hypothesis.

In direct continuity of this work, it would be interesting to investigate the behavior of prediction accuracy on real human data where *n* ≃ *p*. This would enable us to determine whether our approximations still hold in that case, and even in the case where *n* > *p* (where we approximate the empirical covariance matrix of the markers to be diagonal). It would show whether it is possible for the prediction accuracy to exceed the upper bound proposed by De los Campos et al. (2013). A further prospect would be to consider a nonlinear model extension via kernel ridge regression, which may improve the prediction (Morota and Gianola, 2014).

## Data Availability Statement

The data analyzed in this study is subject to the following licenses/restrictions: There is a charge for access to the UK Biobank dataset. Requests to access these datasets should be directed to: https://www.ukbiobank.ac.uk/.

## Ethics Statement

The studies involving human participants were reviewed and approved by NHS National Research Ethics Service North West (11/NW/0382). The patients/participants provided their written informed consent to participate in this study. Written informed consent was obtained from the individual(s) for the publication of any potentially identifiable images or data included in this article.

## Author Contributions

EL and CA designed and directed the study. AF, EL, and CA wrote the manuscript. AF created the synthetic datasets. AF performed all the analyses with substantial input from EL, CA, CD-R, and MP-J. All authors discussed the results and commented on the manuscript.

## Conflict of Interest

The authors declare that the research was conducted in the absence of any commercial or financial relationships that could be construed as a potential conflict of interest.
