# Supplementary material for: Exploring the Link Between Additive Heritability and Prediction Accuracy From a Ridge Regression Perspective
Source: Front Genet. 2020 Nov 4;11:581594. doi: 10.3389/fgene.2020.581594 (PMC7672157; doi:10.3389/fgene.2020.581594)
Supplement: Supplementary file 1 [file Data_Sheet_1.PDF]

Supplementary Material

1 A USEFUL ALGEBRA FOR RIDGE REGRESSION

$$\mathbf{Z}^T\left(\mathbf{Z}\mathbf{Z}^T+\lambda\mathbf{I}_p\right)=\left(\mathbf{Z}^T\mathbf{Z}+\lambda\mathbf{I}_n\right)\mathbf{Z}^T$$

$$\Rightarrow\left(\mathbf{Z}^T\mathbf{Z}+\lambda\mathbf{I}_n\right)^{-1}\mathbf{Z}^T\left(\mathbf{Z}\mathbf{Z}^T+\lambda\mathbf{I}_p\right)\left(\mathbf{Z}\mathbf{Z}^T+\lambda\mathbf{I}_p\right)^{-1}=\left(\mathbf{Z}^T\mathbf{Z}+\lambda\mathbf{I}_n\right)^{-1}\left(\mathbf{Z}^T\mathbf{Z}+\lambda\mathbf{I}_n\right)\mathbf{Z}^T\left(\mathbf{Z}\mathbf{Z}^T+\lambda\mathbf{I}_p\right)^{-1}$$

$$\Rightarrow\left(\mathbf{Z}^T\mathbf{Z}+\lambda\mathbf{I}_n\right)^{-1}\mathbf{Z}^T=\mathbf{Z}^T\left(\mathbf{Z}\mathbf{Z}^T+\lambda\mathbf{I}_p\right)^{-1}.$$

2 COMPUTATION OF THE GCV

2.1 Computation of the LOO error

To compute the leave-one-out error (LOO) error, the estimation of ridge regression parameters without individual i, $\hat{u}_R^{-i}$, is required. Let us recall the Sherman-Morrison-Woodbury's formula : let $\mathbf{A}\in\mathcal{M}_p$ a non-singular matrix and $u, v\in\mathbb{R}^p$.

$$\left(\mathbf{A}+uv^T\right)^{-1}=\mathbf{A}^{-1}-\frac{\mathbf{A}^{-1}uv^T\mathbf{A}^{-1}}{1+v^T\mathbf{A}^{-1}u}. \tag{S1}$$

Using Sherman-Morrison-Woodbury's formula in the context of ridge regression, we have

$$\begin{aligned}\left(\mathbf{Z}_{-i}^T\mathbf{Z}_{-i}+\lambda\mathbf{I}_p\right)^{-1}&=\left(\mathbf{Z}^T\mathbf{Z}-z_iz_i^T+\lambda\mathbf{I}_p\right)^{-1}\\&=\left(\mathbf{Z}^T\mathbf{Z}+\lambda\mathbf{I}_p\right)^{-1}+\frac{\left(\mathbf{Z}^T\mathbf{Z}+\lambda\mathbf{I}_p\right)^{-1}z_iz_i^T\left(\mathbf{Z}^T\mathbf{Z}+\lambda\mathbf{I}_p\right)^{-1}}{1-z_i^T\left(\mathbf{Z}^T\mathbf{Z}+\lambda\mathbf{I}_p\right)^{-1}z_i}\end{aligned}$$

with $z_i\in\mathbb{R}^p$ the column vector corresponding to the normalized genotypes of the i-th row (i.e. the i-th individual) of $\mathbf{Z}$ and $\mathbf{Z}_{-i}\in\mathcal{M}_{n-1,p}(\mathbb{R})$ the matrix $\mathbf{Z}$ excluding its i-th row . Noticing that

$$\mathbf{Z}_{-i}^T\mathbf{y}_{-i}=\mathbf{Z}^T\mathbf{y}-z_iy_i,$$

it is straightforward to get

$$\begin{aligned}\hat{u}_R^{-i}&=\left(\mathbf{Z}_{-i}^T\mathbf{Z}_{-i}+\lambda\mathbf{I}_p\right)^{-1}\mathbf{Z}_{-i}^T\mathbf{y}_{-i}\\&=\left(\left(\mathbf{Z}^T\mathbf{Z}+\lambda\mathbf{I}_p\right)^{-1}+\frac{\left(\mathbf{Z}^T\mathbf{Z}+\lambda\mathbf{I}_p\right)^{-1}z_iz_i^T\left(\mathbf{Z}^T\mathbf{Z}+\lambda\mathbf{I}_p\right)^{-1}}{1-z_i^T\left(\mathbf{Z}^T\mathbf{Z}+\lambda\mathbf{I}_p\right)^{-1}z_i}\right)\left(\mathbf{Z}^T\mathbf{y}-z_iy_i\right)\\&=\hat{u}_R-\left(\mathbf{Z}^T\mathbf{Z}+\lambda\mathbf{I}_p\right)^{-1}z_iy_i+\frac{\left(\mathbf{Z}^T\mathbf{Z}+\lambda\mathbf{I}_p\right)^{-1}z_iz_i^T\left(\mathbf{Z}^T\mathbf{Z}+\lambda\mathbf{I}_p\right)^{-1}}{1-z_i^T\left(\mathbf{Z}^T\mathbf{Z}+\lambda\mathbf{I}_p\right)^{-1}z_i}\left(\mathbf{Z}^T\mathbf{y}-z_iy_i\right)\end{aligned}$$

Using that $z_i^T \left(\mathbf{Z}^T\mathbf{Z} + \lambda\mathbf{I}_p\right)^{-1} z_i = [h_\lambda]_{ii}$ and remembering that y_i is a scalar we have

$$\hat{u}_R^{-i} = \hat{u}_R - \frac{(\mathbf{Z}^T\mathbf{Z} + \lambda\mathbf{I}_p)^{-1} z_i (y_i - z_i^T \hat{u}_R)}{1 - [h_\lambda]_{ii}}.$$

Injecting this expression in the classic Mean Squared Error (MSE), the LOO error expresses as

$$\text{err}^{LOO}(\lambda) = \frac{1}{n}\sum_{i=1}^{n} \left(y_i - z_i^T \hat{u}_R^{-i}(\lambda)\right)^2 \tag{S2}$$

$$= \frac{1}{n}\sum_{i=1}^{n} \left(\frac{y_i - z_i^T \hat{u}_R(\lambda)}{1 - [h_\lambda]_{ii}}\right)^2 \tag{S3}$$

$$= \frac{1}{n}\mathbf{y}^T(\mathbf{I}_n - \mathbf{H}_\lambda)(\text{diag}(\mathbf{I}_n - \mathbf{H}_\lambda))^{-2}(\mathbf{I}_n - \mathbf{H}_\lambda)\mathbf{y}, \tag{S4}$$

where $\mathbf{H}_\lambda = \mathbf{Z}\mathbf{Z}^T(\mathbf{Z}\mathbf{Z}^T + \lambda\mathbf{I}_n)^{-1} = \mathbf{Z}(\mathbf{Z}^T\mathbf{Z} + \lambda\mathbf{I}_p)^{-1}\mathbf{Z}^T$ is the so-called hat matrix because it transforms $\mathbf{y}$ into $\hat{\mathbf{y}}$:

$$\hat{\mathbf{y}} = \mathbf{H}_\lambda \mathbf{y} = \mathbf{Z}\hat{u}_R(\lambda).$$

An important point to notice is that this LOO is not a "true" n-fold cross validation because we standardize our data only once. For the more classical n-fold cross validation we would standardize each training set separately and use this standardization on the validation sample. While it may not look significant, we will show later that this unique standardization has important consequences.

2.2 Computation of the GCV error

Generalized Cross validation (GCV) is an approximation of the LOO. First we introduce some notion about circulant matrices.

A matrix $\mathbf{C}$ is called a circulant matrix if it is of the form

$$\mathbf{C} = \begin{pmatrix} c_0 & c_1 & c_2 & \cdots & c_{n-1} \\ c_{n-1} & c_0 & c_1 & & c_{n-2} \\ c_{n-2} & c_{n-1} & c_0 & & c_{n-3} \\ \vdots & & & \ddots & \vdots \\ c_1 & c_2 & c_3 & \cdots & c_0 \end{pmatrix} \in \text{Circ}(n).$$

Such a matrix has constant diagonal coefficients. Let $\mathbf{W} \in \mathcal{O}_n(\mathbb{C})$ an orthogonal matrix such as $[\mathbf{W}]_{jk} = \frac{1}{\sqrt{n}} e^{2\pi i jk/n}$ with $j, k \in \{1, ..., n\}$. Then $\mathbf{W}$ diagonalize all circulant matrices i.e.

$$\forall \mathbf{C} \in \text{Circ}(n),\ \exists \boldsymbol{\mathcal{D}} \in \mathbb{D}_n(\mathbb{C})\ /\ \mathbf{C} = \mathbf{W}\boldsymbol{\mathcal{D}}\mathbf{W}^*$$

with * the complex transpose operator.

The idea underlying GCV is to project the initial model in a well-chosen complex space such that the matrix $\mathbf{H}_\lambda \in \mathrm{Circ}(n)$. In this new model it is straightforward to compute the inverse of diag$(\mathbf{I}_n - \mathbf{H}_\lambda)$ needed in (S4). This will shorten the computational time.

Let $\mathbf{Z} = \mathbf{U}\mathbf{D}\mathbf{V}^T$ be the singular value decomposition (SVD) of $\mathbf{Z}$ with $\mathbf{U} \in \mathcal{O}_n(\mathbb{R})$, $\mathbf{V} \in \mathcal{O}_p(\mathbb{R})$ and $\mathbf{D} \in \mathcal{M}_{n,p}(\mathbb{R})$ a rectangular matrix with singular values on the diagonal. Using left-multiplication of the initial model by $\mathbf{W}\mathbf{U}^T$

$$\mathbf{W}\mathbf{U}^T\mathbf{y} = \mathbf{W}\mathbf{U}^T\mathbf{Z}u + \mathbf{W}\mathbf{U}^T\mathbf{e}.$$

Since $\mathbf{U} \in \mathcal{O}_n(\mathbb{R})$ and $\mathbf{W} \in \mathcal{O}_n(\mathbb{C})$ one can write

$$\begin{aligned}\left\|\mathbf{W}\mathbf{U}^T\mathbf{y} - \mathbf{W}\mathbf{U}^T\mathbf{Z}u\right\|_2^2 + \lambda\|u\|_2^2 &= (\mathbf{y} - \mathbf{Z}u)^T\,\mathbf{U}\mathbf{W}^*\mathbf{W}\mathbf{U}^T(\mathbf{y} - \mathbf{Z}u) + \lambda\|u\|_2^2 \\ &= \|\mathbf{y} - \mathbf{Z}u\|_2^2 + \lambda\|u\|_2^2\end{aligned}$$

so λ_{opt} is the same in the two models.

The hat matrix in this new model is

$$\begin{aligned}\tilde{\mathbf{H}}_\lambda &= (\mathbf{W}\mathbf{U}^T\mathbf{Z})(\mathbf{W}\mathbf{U}^T\mathbf{Z})^*\left((\mathbf{W}\mathbf{U}^T\mathbf{Z})(\mathbf{W}\mathbf{U}^T\mathbf{Z})^* + \lambda\mathbf{I}_n\right)^{-1} \\ &= (\mathbf{W}\mathbf{D}\mathbf{V}^T)(\mathbf{W}\mathbf{D}\mathbf{V}^T)^*\left((\mathbf{W}\mathbf{D}\mathbf{V}^T)(\mathbf{W}\mathbf{D}\mathbf{V}^T)^* + \lambda\mathbf{W}\mathbf{W}^*\right)^{-1} \\ &= \mathbf{W}\mathbf{D}\mathbf{D}^T(\mathbf{D}\mathbf{D}^T + \lambda\mathbf{I}_n)^{-1}\mathbf{W}^* \in \mathrm{Circ}(n)\end{aligned}$$

We showed that $\tilde{\mathbf{H}}_\lambda \in \mathrm{Circ}(n)$ and we approximate $\forall i \in [\![1, n]\!],\ [h_\lambda]_{ii}$ by

$$\frac{1}{n}\mathrm{tr}(\tilde{\mathbf{H}}_\lambda) = \frac{1}{n}\sum_{k=1}^{n}\frac{d_k^2}{d_k^2 + \lambda} = \frac{1}{n}\mathrm{tr}(\mathbf{H}_\lambda).$$

Applying this in the expression of err^{LOO} we have

$$\begin{aligned}\mathrm{err}^{GCV} &= \frac{\left\|\mathbf{W}\mathbf{U}^T\mathbf{y} - \tilde{\mathbf{H}}_\lambda\mathbf{y}\right\|_2^2}{\left[\frac{1}{n}\mathrm{tr}\left(\mathbf{I}_n - \tilde{\mathbf{H}}_\lambda\right)\right]^2} \\ &= \frac{\|\mathbf{y} - \hat{\mathbf{y}}(\lambda)\|_2^2}{\left[\frac{1}{n}\mathrm{tr}\left(\mathbf{I}_n - \mathbf{H}_\lambda\right)\right]^2}.\end{aligned}$$

3 PRACTICAL CHOICE OF λ

3.1 A grid of λ for GCV

An important issue in ridge regression is the search for the optimal λ. A grid of λ is often chosen empirically. In the context of the additive polygenic model, it is possible to use the link between heritability

and λ to determine a grid of λ:

$$\underbrace{\{0.01, 0.02, ..., 0.99\}}_{h_G^2} \rightarrow \underbrace{\{p\frac{1-0.01}{0.01}, p\frac{1-0.02}{0.02}, ..., p\frac{1-0.99}{0.99}\}}_{\lambda}.$$

3.2 Using Singular Value Decomposition to speed-up GCV computation

Applying GCV with $n > p$, we would compute $\hat{u}_R = (\mathbf{Z}^T\mathbf{Z} + \lambda\mathbf{I}_p)^{-1}\mathbf{Z}^T\mathbf{y}$ using for each λ and use it to make prediction. In the context of GWAS (i.e. $p >> n$), this is not optimal since it implies the inversion of a $p \times p$ matrix. In our situation, the dual solution of ridge regression is much more adapted, leading to:

$$\hat{\mathbf{y}}(\lambda) = \mathbf{Z}\hat{u}_R(\lambda) = \mathbf{Z}\mathbf{Z}^T\left(\mathbf{Z}\mathbf{Z}^T + \lambda\mathbf{I}_n\right)^{-1}\mathbf{y} = \mathbf{H}_\lambda\mathbf{y}. \tag{S5}$$

GCV can be rewritten for more efficient computation. Let $\mathbf{Z} = \mathbf{U}\mathbf{D}\mathbf{V}^T$ be the singular value decomposition (SVD) of $\mathbf{Z}$ with $\mathbf{U} \in \mathcal{O}_n(\mathbb{R})$, $\mathbf{V} \in \mathcal{O}_p(\mathbb{R})$ and $\mathbf{D} \in \mathcal{M}_{n,p}(\mathbb{R})$ a rectangular matrix with singular values on the diagonal, we have $\mathbf{Z}\mathbf{Z}^T = \mathbf{U}\mathbf{D}\mathbf{D}^T\mathbf{U}^T$ the eigen decomposition of $\mathbf{Z}\mathbf{Z}^T$. Rewriting $\mathbf{H}_{\boldsymbol{\lambda}}$ using the SVD and applying it to GCV leads to:

$$\mathbf{H}_{\boldsymbol{\lambda}} = \mathbf{U}\left[\mathbf{D}\mathbf{D}^T\left(\mathbf{D}\mathbf{D}^T + \lambda\mathbf{I}_n\right)^{-1}\right]\mathbf{U}^T = \mathbf{U}\boldsymbol{\mathcal{D}}_\lambda\mathbf{U}^T, \tag{S6}$$

$$\text{err}^{GCV} = \mathbf{b}^T\left(\mathbf{I}_n - \boldsymbol{\mathcal{D}}_\lambda\right)\left[\frac{1}{n}\text{tr}\left(\mathbf{I_n} - \boldsymbol{\mathcal{D}}_\lambda\right)\mathbf{I_n}\right]^{-2}\left(\mathbf{I}_n - \boldsymbol{\mathcal{D}}_\lambda\right)\mathbf{b},\ \mathbf{b} = \mathbf{U}^T\mathbf{y} \tag{S7}$$

Assuming that we have access to the eigen-decomposition of $\mathbf{Z}\mathbf{Z}^T$, the GCV computation as a function of diagonal matrices is extremely efficient. The most time-consuming part is the eigen decomposition (or the SVD).

4 ISSUE WITH EMPIRICAL SCALING IN THE HIGH DIMENSIONAL CASE

In this section we highlight the issue of the LOO / GCV with "naive" estimation of the intercept using empirically scaled matrices in the high dimensional context ($n < p$). We first show why LOO does not work in this setup, then show why GCV does not work either and briefly highlight the issue for the "naive" estimation of more general fixed effects.

In the following, we still consider the genotype matrix $\mathbf{Z}$ and the phenotype vector $\mathbf{y}$ to be empirically scaled. One immediate consequence of this scaling is $\forall i \in [\![1, n]\!], y_i = -\sum_{j\neq i} y_j$ and $z_i = -\sum_{j\neq i} z_j$. Since each row of $\mathbf{Z}$ is a linear combination of the others, we also have $0 \in \text{sp}\left(\mathbf{Z}\mathbf{Z}^T\right)$.

4.1 Constant eigenvectors associated with the null eigenvalue

Let $\mathbf{w} = \alpha\mathbb{1}_{\boldsymbol{n}}$ with $\alpha \in \mathbb{R}$. Since $\mathbf{Z}$ is normalized with the empirical scaling $\mathbf{Z}^T\mathbf{w} = 0_p \rightarrow \mathbf{Z}\mathbf{Z}^T\mathbf{w} = \mathbf{0}_{\boldsymbol{n}} = 0\mathbf{w}$ so the eigenvectors associated with 0 are constant.

Since we choose this eigenvector to have a unit norm, we have $\|\mathbf{w}\|_2^2 = \alpha^2\,\|\mathbb{1}_{\boldsymbol{n}}\|_2^2 = \alpha^2 n$. In the end, $\mathbf{w} = \frac{1}{\sqrt{n}}\mathbb{1}_{\boldsymbol{n}}$ or $\mathbf{w} = \frac{-1}{\sqrt{n}}\mathbb{1}_{\boldsymbol{n}}$.

4.2 LOO standardization problem in a high dimensional setting

Let $\mathbf{Z}_{-i} = \begin{pmatrix} z_1 \\ \vdots \\ z_{i-1} \\ z_{i+1} \\ \vdots \\ z_n \end{pmatrix} \in \mathcal{M}_{n-1,p}(\mathbb{R})$, $\mathbf{y}_{-i} = \begin{pmatrix} y_1 \\ \vdots \\ y_{i-1} \\ y_{i+1} \\ \vdots \\ y_n \end{pmatrix}$ and $\hat{u}_R^{-i} = \mathbf{Z}_{-i}^T \left(\mathbf{Z}_{-i}\mathbf{Z}_{-i}^T + \lambda \mathbf{I}_{n-1} \right)^{-1} \mathbf{y}_{-i}$.

Then, we have $\hat{\mathbf{y}}_{-i}(i) = z_i^T \hat{u}_R^{-i} = -\sum_{j \neq i} z_j^T \hat{u}_R^{-i} = -\mathbb{1}_n^T \mathbf{Z}_{-i}\mathbf{Z}_{-i}^T \left(\mathbf{Z}_{-i}\mathbf{Z}_{-i}^T + \lambda \mathbf{I}_{n-1} \right)^{-1} \mathbf{y}_{-i}$.

We assume the variants to be independent and can reasonably suppose that the individuals are linearly independent when $n < p$. In that case $\mathbf{Z}_{-i}\mathbf{Z}_{-i}^T$ is invertible in spite of empirical centering because the empirical centering includes the i-th individual. We notice that when $\lambda \to 0$, $\hat{\mathbf{y}}_{-i}(i) \to -\sum_{j \neq i} y_j = y_i$. Then, we easily show that $\left(\hat{\mathbf{y}}_{-i}(i) - y_i \right)^2 \to 0$ and $\text{err}^{LOO} \underset{\lambda \to 0}{\to} 0$.

Here we see the influence from the unique standardisation of this LOO : because we used all individuals for standardization a phenomenon of dependency appears between the training and validation sets. Have we used a classical n-fold cross validation we would not have such dependencies, since the standardization would only include the training set.

4.3 GCV standardization problem in a high dimensional setting

Starting from GCV formula and assuming $n < p$

$$\begin{aligned} \text{err}^{GCV}(\mathbf{y}, \mathbf{Z}, \lambda) &= \frac{1}{n} \mathbf{y}^T (\mathbf{I}_n - \mathbf{H}_\lambda) \left[\frac{1}{n} \text{tr}(\mathbf{I}_n - \mathbf{H}_\lambda) \mathbf{I}_n \right]^{-2} (\mathbf{I}_n - \mathbf{H}_\lambda) \mathbf{y} \\ &= \frac{1}{n} \mathbf{b}^T (\mathbf{I}_n - \boldsymbol{\mathcal{D}}_\lambda) \left[\frac{1}{n} \text{tr}(\mathbf{I}_n - \boldsymbol{\mathcal{D}}_\lambda) \mathbf{I}_n \right]^{-2} (\mathbf{I}_n - \boldsymbol{\mathcal{D}}_\lambda) \mathbf{b} \end{aligned}$$

where $\mathbf{b} = \mathbf{U}^T \mathbf{y}$ and $\boldsymbol{\mathcal{D}}_\lambda = \mathbf{D}\mathbf{D}^T \left(\mathbf{D}\mathbf{D}^T + \lambda \mathbf{I}_n \right)^{-1}$.

Let d_n^2 the null eigenvalue of $\mathbf{Z}\mathbf{Z}^T$ thus obtained. Noticing that $\mathbf{I}_n - \boldsymbol{\mathcal{D}}_\lambda \xrightarrow[\lambda \to 0]{d_n^2 = 0} \begin{pmatrix} 0 & & & \\ & \ddots & & \\ & & 0 & \\ & & & 1 \end{pmatrix}$, we have $\left[\frac{1}{n} \text{tr}(\mathbf{I}_n - \boldsymbol{\mathcal{D}}_\lambda) \mathbf{I}_n \right]^{-2} \xrightarrow[\lambda \to 0]{d_n^2 = 0} n^2 \mathbf{I}_n$.

We then have

$$\text{err}^{GCV}(\mathbf{y}, \mathbf{Z}, \lambda) \xrightarrow[\lambda \to 0]{d_n^2 = 0} \frac{1}{n} \times n^2 \mathbf{b}^T \begin{pmatrix} 0 & & & \\ & \ddots & & \\ & & 0 & \\ & & & 1 \end{pmatrix} \mathbf{b} = n b_n^2.$$

Using 4.1, $b_n^2 = \frac{1}{n}(\mathbb{1}_{\boldsymbol{n}}{}^T\mathbf{y})^2$ and so

$$\text{err}^{GCV}(\mathbf{y}, \mathbf{Z}, \lambda) \xrightarrow[\lambda\to 0]{d_n^2=0} (\mathbb{1}_{\boldsymbol{n}}{}^T\mathbf{y})^2 = 0.$$

A similar issue can be observed in the presence of covariates. Let $\mathbf{X} \in \mathcal{M}_{n,r}(\mathbb{R})$ the empirically scaled matrix of covariates. A "naive" approach to take into account those covariates would be to perform linear regression of the phenotypes (which we assumed to be centered) on the empirically scaled covariates and then to apply GCV on the residuals. Let $\hat{\beta}$ the least square estimator, in this setup

$$\begin{aligned}\text{err}^{GCV}(\mathbf{y} - \mathbf{X}\hat{\beta}, \mathbf{Z}, \lambda) &\xrightarrow[\lambda\to 0]{d_n^2=0} (\mathbb{1}_{\boldsymbol{n}}{}^T\mathbf{y} - \mathbb{1}_{\boldsymbol{n}}{}^T\mathbf{X}\hat{\beta})^2 \\ &= (0-0)^2 \\ &= 0.\end{aligned}$$

5 PROJECTION-BASED APPROACH FOR GCV WITH COVARIATES USING QR DECOMPOSITION

QR decomposition allows an easy construction of a contrast matrix. The QR decomposition of $\mathbf{A} \in \mathbb{M}_{n,r}(\mathbb{R})$ is $\mathbf{A} = \mathbf{QR}$ with $\mathbf{Q} \in \mathcal{O}(n)$ and $\mathbf{R}^T \in \mathcal{M}_{r,n}(\mathbb{R}) = [\mathbf{R}_1^T, \mathbf{0}_{r,n-r}]$ where $\mathbf{R}_1 \in \mathcal{M}_{r,r}(\mathbb{R})$ is an upper triangular matrix.

Let $\mathbf{Q} = [\mathbf{Q}_1, \mathbf{Q}_2]$ with $\mathbf{Q}_1 \in \mathcal{M}_{n,r}(\mathbb{R}),\ \mathbf{Q}_2 \in \mathcal{M}_{n,n-r}(\mathbb{R})$ and observing that

$$\mathbf{Q}^T\mathbf{Q} = \begin{pmatrix}\mathbf{Q}_1^T \\ \mathbf{Q}_2^T\end{pmatrix}\begin{pmatrix}\mathbf{Q}_1 & \mathbf{Q}_2\end{pmatrix} = \begin{pmatrix}\mathbf{Q}_1^T\mathbf{Q}_1 & \mathbf{Q}_1^T\mathbf{Q}_2 \\ \mathbf{Q}_2^T\mathbf{Q}_1 & \mathbf{Q}_2^T\mathbf{Q}_2\end{pmatrix} = \begin{pmatrix}\mathbf{I}_r & \mathbf{O}_{r,n-r} \\ \mathbf{O}_{n-r,r} & \mathbf{I}_{n-r}\end{pmatrix},$$

we can show

$$\mathbf{Q}_2^T\mathbf{A} = \mathbf{Q}_2^T\begin{pmatrix}\mathbf{Q}_1 & \mathbf{Q}_2\end{pmatrix}\mathbf{R} = \begin{pmatrix}\mathbf{0}_{n-r,r} & \mathbf{I}_{n-r}\end{pmatrix}\begin{pmatrix}\mathbf{R}_1 \\ \mathbf{0}_{n-r,n}\end{pmatrix} = \mathbf{0}_{n-r,r}.$$

$\mathbf{Q}_2^T$ is a contrast matrix since we have $\mathbf{Q}_2^T\mathbf{A} = \mathbf{0}_{n-r,r}$ and $\mathbf{Q}_2^T\mathbf{Q}_2 = \mathbf{I}_{n-r}$. The QR decomposition of a matrix being relatively inexpensive to compute, this proposed method offers an interesting alternative.

6 LINK BETWEEN RANDOM EFFECTS MODEL AND RIDGE REGRESSION

6.1 The case without fixed effects

Ridge regression and random effects model are closely linked. Starting by the maximizing the posterior of the parameters of

$$\mathbf{y} = \mathbf{Z}u + \mathbf{e}$$

where $u \sim \mathcal{N}(0_p, \tau \mathbf{I}_p)$ and $\mathbf{e} \sim \mathcal{N}(0_n, \sigma^2 \mathbf{I}_n)$. Our goal is to maximize

$$\begin{aligned} p(u|\mathbf{y}) &= \frac{p(\mathbf{y}|u)p(u)}{p(\mathbf{y})} \\ \rightarrow \log p(u|\mathbf{y}) &= \log p(\mathbf{y}|u) + \log p(u) - \log p(\mathbf{y}). \end{aligned}$$

Using the fact that $u \sim \mathcal{N}(0_p, \tau \mathbf{I}_p)$, $\mathbf{y} \sim \mathcal{N}(\mathbf{0}_n, \tau \mathbf{Z}\mathbf{Z}^T + \sigma^2 \mathbf{I}_n)$, $\mathbf{y}|u \sim \mathcal{N}(\mathbf{Z}u, \sigma^2 \mathbf{I}_n)$ and remembering the formula of the log-likelihood for a gaussian distribution of parameters μ and $\mathbf{\Sigma}$ is

$$\log p(\mathbf{x}|\mu, \mathbf{\Sigma}) = -\frac{n}{2}\log 2\pi - \frac{1}{2}\log |\mathbf{\Sigma}| - (\mathbf{x} - \mu)^T \mathbf{\Sigma}^{-1} (\mathbf{x} - \mu),$$

we can write

$$\begin{aligned} \log p(u|\mathbf{y}) = &- \frac{n}{2}\log 2\pi - \frac{1}{2}\log \left|\sigma^2 \mathbf{I}_n\right| - \frac{1}{2}(\mathbf{y} - \mathbf{Z}u)^T (\sigma^2 \mathbf{I}_n)^{-1} (\mathbf{y} - \mathbf{Z}u) \\ &- \frac{p}{2}\log 2\pi - \frac{1}{2}\log |\tau \mathbf{I}_p| - \frac{1}{2}(u - 0_p)^T (\tau \mathbf{I}_p)^{-1} (u - 0_p) \\ &+ \frac{n}{2}\log 2\pi + \frac{1}{2}\log \left|\tau \mathbf{Z}\mathbf{Z}^T + \sigma^2 \mathbf{I}_n\right| - \frac{1}{2}(\mathbf{y} - \mathbf{0}_n)^T (\tau \mathbf{Z}\mathbf{Z}^T + \sigma^2 \mathbf{I}_n)^{-1} (\mathbf{y} - \mathbf{0}_n). \end{aligned}$$

By isolating the terms dependent on u, we obtain

$$\log p(u|\mathbf{y}) = -\frac{1}{2\sigma^2}\left(\|\mathbf{y} - \mathbf{Z}u\|_2^2 + \frac{\sigma^2}{\tau}\|u\|_2^2\right) + K_{\perp u}$$

with $K_{\perp u}$ a term independent of u. After simplification we have

$$\arg\max_u p\,(u|\mathbf{y}) = \arg\min_u \|\mathbf{y} - \mathbf{Z}u\|_2^2 + \lambda \|u\|_2^2 \text{ with } \lambda = \frac{\sigma^2}{\tau}. \tag{S8}$$

6.2 An extension for the mixed model

It is also possible to exhibit a link between mixed model (that is a random effects model with additional covariates with non-random effects) and ridge regression with some covariates we do not wish to penalize. Assuming the following model:

$$\mathbf{y} = \mathbf{X}\beta + \mathbf{Z}u + \mathbf{e}$$

and denoting $\mathbf{C}$ a contrast matrix such that $\mathbf{CX} = \mathbf{0}_{n,r}$ and $\mathbf{CC}^T = \mathbf{I}_{n-r}$.

The left multiplication of the above by $\mathbf{C}$ gives

$$\mathbf{Cy} = \mathbf{CX}\beta + \mathbf{CZ}u + \mathbf{Ce} = \mathbf{CZ}u + \mathbf{Ce}.$$

Noticing that $\mathbf{Cy}|u \sim \mathcal{N}(\mathbf{CZ}u, \sigma^2\mathbf{I}_{n-r})$ and $\mathbf{Cy} \sim \mathcal{N}(\mathbf{0}_{n-r}, \tau\mathbf{CZZ}^T\mathbf{C}^T + \sigma^2\mathbf{I}_{n-r})$ we can write the posterior of the contrasted model as

$$\log p(u|\mathbf{Cy}) = -\frac{1}{2}\left(\|\mathbf{Cy} - \mathbf{CZ}u\|_2^2 + \frac{\sigma}{\tau}\|u\|_2^2\right) + K_{\perp u}$$

and after simplification

$$\arg\max_u p\,(u|\mathbf{Cy}) = \arg\min_u \|\mathbf{Cy} - \mathbf{CZ}u\|_2^2 + \lambda\,\|u\|_2^2 \text{ with } \lambda = \frac{\sigma^2}{\tau}.$$

7 THE PROPORTION OF CAUSAL VARIANTS DOES NOT IMPACT HERITABILITY ESTIMATION

7.1 Estimation of heritability on synthetic data

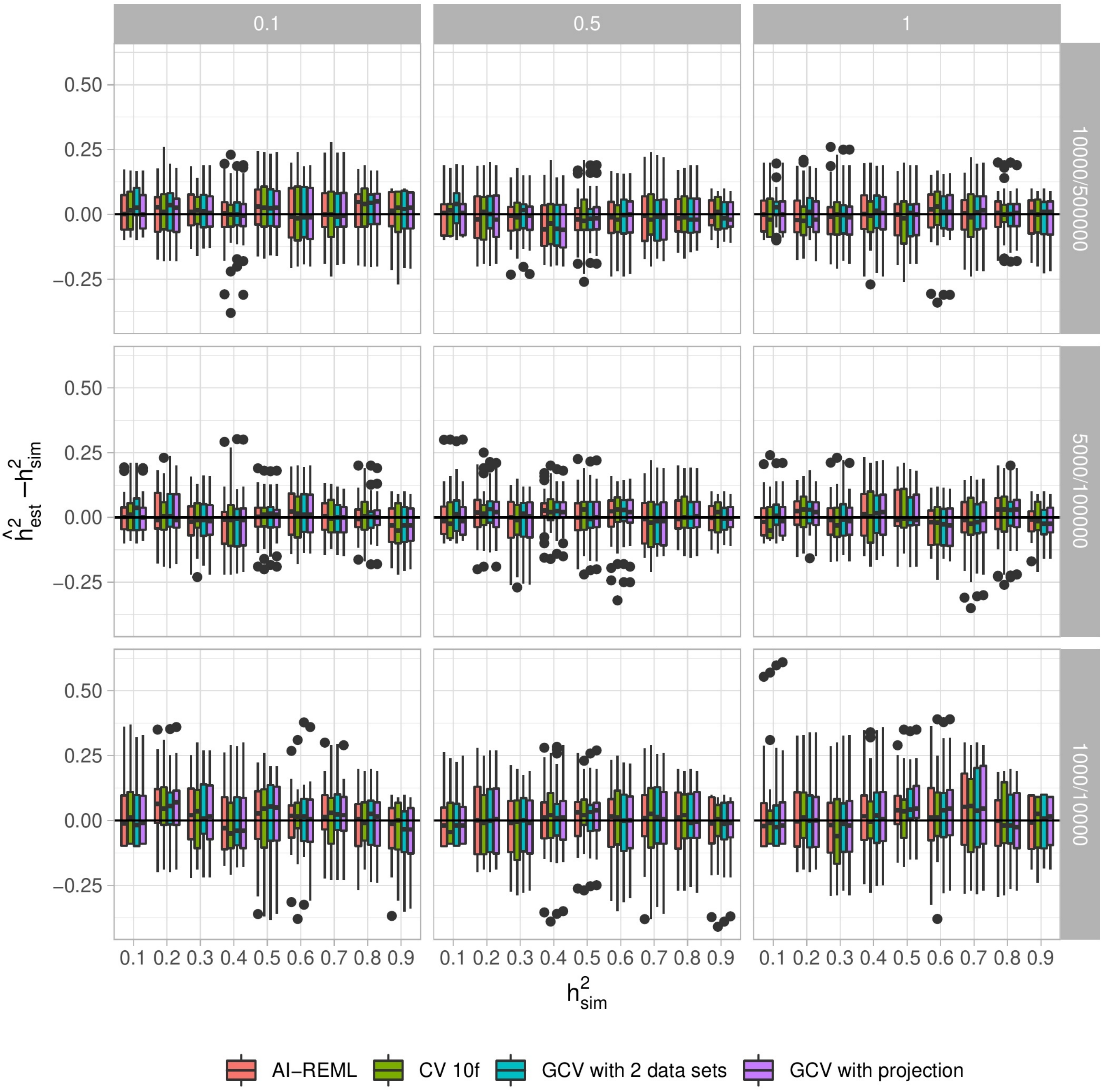

Figure S1: Distribution of $(h^2_{est} - h^2_{sim})$ for multiple parameter combinations with 30 replications. Data are simulated under the fully synthetic procedure. The columns of the grid correspond to the fraction of causal variants while the lines correspond to the ratio n/p. For each panel the horizontal axis corresponds to the simulated heritability $h^2_{sim} \in \{0.1, ..., \}$ and the vertical axis corresponds to the estimation of $h^2_g - h^2_{sim}$. Heritability estimations are done with random effects model using AI-REML to estimate the variance components and with ridge regression using 3 approaches for the choice of λ_{opt} : GCV with a projection correction and GCV with a 2nd dataset correction and a 10 fold cross validation (Ridge 10fCV).

7.2 Estimation of heritability on semi-synthetic data

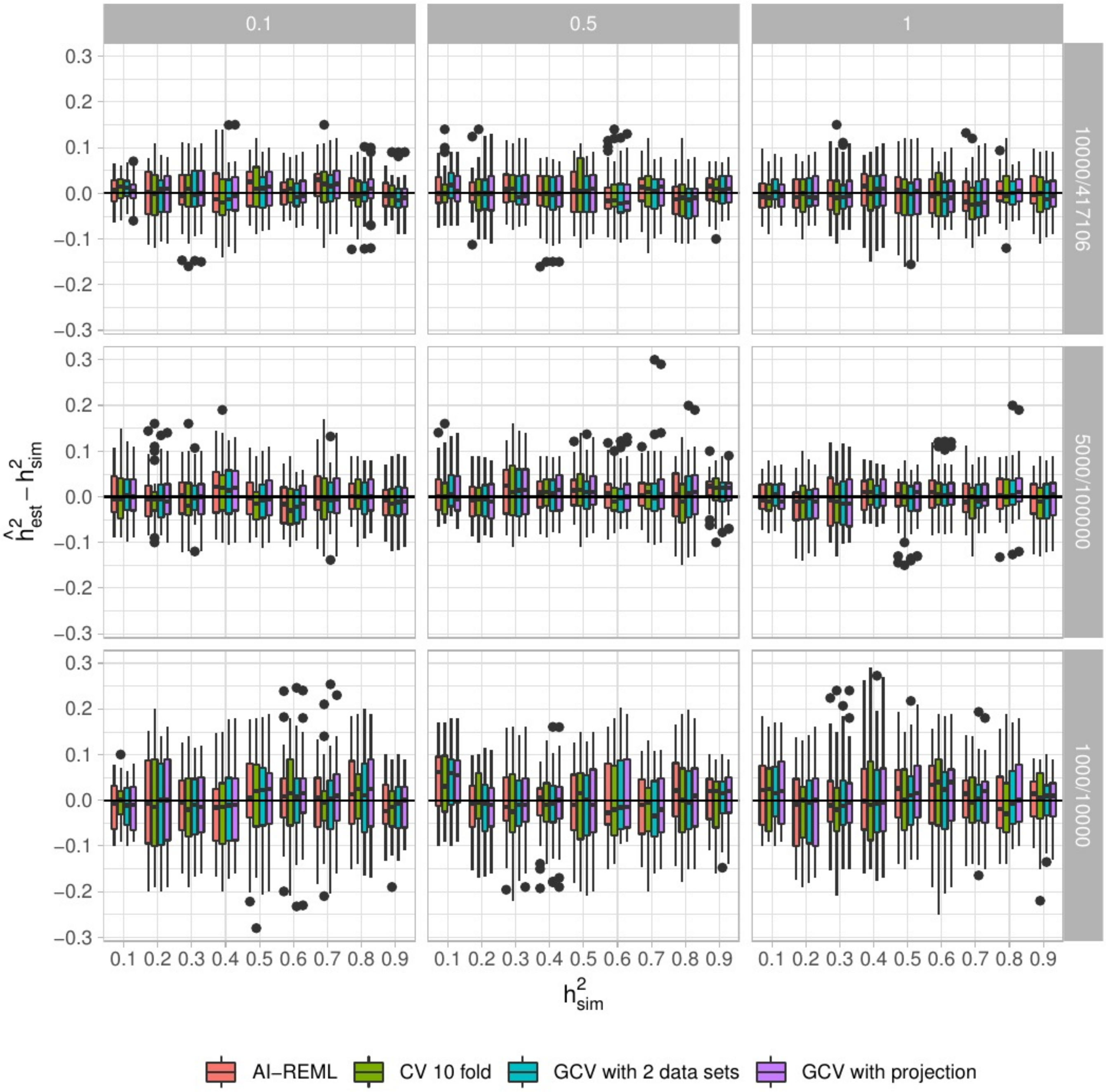

Figure S2: Distribution of $(h^2_{est} - h^2_{sim})$ for multiple parameter combinations with 30 replications. Data are simulated under the semi-synthetic procedure. The columns of the grid correspond to the fraction of causal variants while the lines correspond to the ratio n/p. For each panel the horizontal axis corresponds to the simulated heritability $h^2_{sim} \in \{0.1, ..., \}$ and the vertical axis corresponds to the estimation of $h^2_g - h^2_{sim}$. Heritability estimations are done with random effects model using AI-REML to estimate the variance components and with ridge regression using 3 approaches for the choice of λ_{opt}: GCV with a projection correction and GCV with a 2nd dataset correction and a 10 fold cross validation (Ridge 10fCV).

8 APPROXIMATION OF PREDICTIVE POWER

In this section we detail our approximation of the MSE and squared correlation. In the following the index $_{tr}$ refers to the training set whereas $_{te}$ refers to the test set. To lighten notations $\mathbf{Z}$ is the normalized genotype matrix of the training set. Let $z_{te} \in \mathbb{R}^p$ the column vector corresponding to the normalized genotypes of one test individual. We assume $\mathbb{E}_{z_{te}}\left[z_{te}\right] = 0_p$ and $\text{var}(z_{te}) = \mathbf{I}_p$.

We remind that $\hat{u} = \mathbf{Z}^T\left(\mathbf{Z}\mathbf{Z}^T + \lambda\mathbf{I}_n\right)^{-1}\mathbf{y}_{tr} = \left(\mathbf{Z}^T\mathbf{Z} + \lambda\mathbf{I}_p\right)^{-1}\mathbf{Z}^T\mathbf{y}_{tr} = \mathbf{K}_\lambda\mathbf{y}_{tr}$. We assume the phenotype to have unit variance without loss of generality.

Lastly we remind that for $\mathbf{x}$ a random vector with $\mathbb{E}[\mathbf{x}] = \mathbf{a}$ and $\text{var}(\mathbf{x}) = \mathbf{V}$ we have for any matrix $\mathbf{B}$ $\mathbb{E}[\mathbf{x}^T\mathbf{B}\mathbf{x}] = \text{tr}(\mathbf{B}\mathbf{V}) + \mathbf{a}^T\mathbf{B}\mathbf{a}$.

8.1 Approximation of the mean squared error on the test set

Using the classic bias-variance decomposition and assuming $\mathbf{Z}$ fixed, we can write

$$\begin{aligned}\mathbb{E}_{\mathbf{y}_{tr},y_{te},z_{te}}\left[(y_{te}-\hat{y}_{te})^2\right] &= \mathbb{E}_{z_{te}}\left[\mathbb{E}_{\mathbf{y}_{tr},y_{te}|z_{te}}\left[(y_{te}-\hat{y}_{te})^2\right]\right] \\ &= \mathbb{E}_{z_{te}}\left[\text{var}(y_{te}|z_{te}) + \text{var}(\hat{y}_{te}|z_{te}) + \left(\mathbb{E}_{\mathbf{y}_{tr}|z_{te}}\left[\hat{y}_{te}\right] - \mathbb{E}_{y_{te}|z_{te}}[y_{te}]\right)^2\right].\end{aligned}$$

Firstly we have

$$\begin{aligned}\mathbb{E}_{y_{te},z_{te}}[y_{te}] &= \mathbb{E}_{y_{te},z_{te}}[z_{te}^T u + e_{te}] = \mathbb{E}_{z_{te}^T}[z_{te}]u + \mathbb{E}_{e_{te}}[e_{te}] = 0. \\ \mathbb{E}_{\mathbf{y}_{tr}}[y_{te}] &= y_{te}. \\ \mathbb{E}_{y_{te},z_{te}}[\hat{y}_{te}] &= \mathbb{E}_{y_{te},z_{te}}[z_{te}^T\mathbf{K}_\lambda\mathbf{y}_{tr}] = 0. \\ \mathbb{E}_{\mathbf{y}_{tr}}[\hat{y}_{te}] &= \mathbb{E}_{\mathbf{y}_{tr}}[z_{te}^T\mathbf{K}_\lambda\mathbf{y}_{tr}] = z_{te}^T\mathbf{K}_\lambda\mathbf{Z}u.\end{aligned}$$

Developing the 3 terms of the bias-variance decomposition using the expected value of a quadratic form we have

$$\begin{aligned}\text{var}(y_{te}|z_{te}) &= \mathbb{E}_{y_{te}|z_{te}}\left[\left(y_{te} - \mathbb{E}_{y_{te}|z_{te}}\left[y_{te}\right]\right)^2\right] = \mathbb{E}_{y_{te}|z_{te}}\left[e_{te}^2\right] = \sigma^2 \\ \text{var}(\hat{y}_{te}|z_{te}) &= \mathbb{E}_{\mathbf{y}_{tr}|z_{te}}\left[\hat{y}_{te}^2\right] - \mathbb{E}_{\mathbf{y}_{tr}|z_{te}}\left[\hat{y}_{te}\right]^2 \\ &= \mathbb{E}_{\mathbf{y}_{tr}|z_{te}}\left[\mathbf{y}_{tr}^T\mathbf{K}_\lambda^T z_{te} z_{te}^T\mathbf{K}_\lambda\mathbf{y}_{tr}\right] - (\mathbf{Z}u)^T\mathbf{K}_\lambda^T z_{te} z_{te}^T\mathbf{K}_\lambda(\mathbf{Z}u) \\ &= \text{tr}\left(\mathbf{K}_\lambda^T z_{te} z_{te}^T\mathbf{K}_\lambda\sigma^2\mathbf{I}_n\right) + (\mathbf{Z}u)^T\mathbf{K}_\lambda^T z_{te} z_{te}^T\mathbf{K}_\lambda(\mathbf{Z}u) \\ &\quad - (\mathbf{Z}u)^T\mathbf{K}_\lambda^T z_{te} z_{te}^T\mathbf{K}_\lambda(\mathbf{Z}u) \\ &= \sigma^2 z_{te}^T\mathbf{K}_\lambda\mathbf{K}_\lambda^T z_{te} \\ \left(\mathbb{E}_{\mathbf{y}_{tr}|z_{te}}\left[\hat{y}_{te}\right] - \mathbb{E}_{y_{te}|z_{te}}[y_{te}]\right)^2 &= \left(z_{te}^T\mathbf{K}_\lambda(\mathbf{Z}u) - z_{te}^T u\right)^2 \\ &= \left(z_{te}^T\left(\mathbf{K}_\lambda\mathbf{Z} - \mathbf{I}_p\right)u\right)^2 \\ &= z_{te}^T\left(\mathbf{K}_\lambda\mathbf{Z} - \mathbf{I}_p\right)uu^T\left(\mathbf{K}_\lambda\mathbf{Z} - \mathbf{I}_p\right)z_{te}.\end{aligned}$$

since $(\mathbf{K}_\lambda \mathbf{Z})^T = (\mathbf{Z}^T(\mathbf{Z}\mathbf{Z}^T + \lambda \mathbf{I}_n)^{-1}\mathbf{Z})^T = \mathbf{Z}^T(\mathbf{Z}\mathbf{Z}^T + \lambda \mathbf{I}_n)^{-1}\mathbf{Z} = \mathbf{K}_\lambda \mathbf{Z}$.

Applying the expectation over z_{te} on those 3 terms

$$
\begin{aligned}
\mathbb{E}_{z_{te}}\left[\text{var}(y_{te}|z_{te})\right] &= \sigma^2 \\
\mathbb{E}_{z_{te}}\left[\text{var}(\hat{y}_{te}|z_{te})\right] &= \sigma^2 \text{tr}\left(\mathbf{K}_\lambda \mathbf{K}_\lambda^T\right) \\
\mathbb{E}_{z_{te}}\left[\left(\mathbb{E}_{\mathbf{y}_{tr}|z_{te}}\left[\hat{y}_{te}\right] - \mathbb{E}_{y_{te}|z_{te}}[y_{te}]\right)^2\right] &= u^T\left(\mathbf{K}_\lambda \mathbf{Z} - \mathbf{I}_p\right)^2 u \\
&= u^T\left(\mathbf{K}_\lambda \mathbf{Z}\mathbf{K}_\lambda \mathbf{Z} - 2\mathbf{K}_\lambda \mathbf{Z} + \mathbf{I}_p\right) u.
\end{aligned}
$$

We first approximate the case $n < p$. Here we can reasonably suppose that $\mathbf{Z}\mathbf{Z}^T \simeq p\mathbf{I}_n$ since we are working on unrelated individuals and because of the normalization of $\mathbf{Z}$. Using this approximation one can write

$$
\mathbf{Z}\mathbf{Z}^T \simeq p\mathbf{I}_n \Rightarrow \mathbf{K}_\lambda \simeq \frac{1}{p+\lambda}\mathbf{Z}^T.
$$

Replacing $\mathbf{Z}\mathbf{Z}^T$ in the above expressions and using the link between ridge regression parameter and heritability we have

$$
\begin{aligned}
\mathbb{E}_{z_{te}}\left[\text{var}(\hat{y}_{te}|z_{te})\right] &= \sigma^2 \text{tr}\left(\mathbf{K}_\lambda \mathbf{K}_\lambda^T\right) \\
&\simeq \sigma^2 \text{tr}\left(\left(\frac{1}{p+\lambda}\mathbf{Z}^T\right)\left(\frac{1}{p+\lambda}\mathbf{Z}^T\right)^T\right) \\
&= \sigma^2\left(\frac{1}{p+\lambda}\right)^2 \text{tr}\left(\mathbf{Z}^T\mathbf{Z}\right) \\
&\simeq \sigma^2\left(\frac{1}{p+\lambda}\right)^2 \text{tr}\left(p\mathbf{I}_n\right) \\
&= \sigma^2\left(\frac{1}{p+\lambda}\right)^2 np \\
&= (1-h^2)(h^2)^2\frac{n}{p}
\end{aligned}
$$

and

$$\begin{aligned}
\mathbb{E}_{z_{te}}\left[\left(\mathbb{E}_{\mathbf{y}_{tr}|z_{te}}\left[\hat{y}_{te}\right]-\mathbb{E}_{y_{te}|z_{te}}[y_{te}]\right)^2\right] &= u^T\left(\mathbf{K}_\lambda\mathbf{Z}-\mathbf{I}_p\right)^2 u\\
&= u^T\left(\left(\frac{1}{p+\lambda}\right)^2\mathbf{Z}^T\mathbf{Z}\mathbf{Z}^T\mathbf{Z}-2\left(\frac{1}{p+\lambda}\right)\mathbf{Z}^T\mathbf{Z}+\mathbf{I}_p\right)u\\
&= p\left(\frac{1}{p+\lambda}\right)^2(\mathbf{Z}u)^T(\mathbf{Z}u)-2\left(\frac{1}{p+\lambda}\right)(\mathbf{Z}u)^T(\mathbf{Z}u)+u^Tu\\
&\simeq p\left(\frac{1}{p+\lambda}\right)^2 nh^2-2\left(\frac{1}{p+\lambda}\right)nh^2+h^2\\
&= \frac{n}{p}(h^2)^3-2\frac{n}{p}(h^2)^2+h^2\\
&= h^2\left(1+\frac{n}{p}\left((h^2)^2-2h^2\right)\right).
\end{aligned}$$

Summing all those expressions, we end up with

$$\begin{aligned}
\mathbb{E}_{\mathbf{y}_{tr},y_{te},z_{te}}\left[(y_{te}-\hat{y}_{te})^2\right] &\simeq 1-h^2+(1-h^2)(h^2)^2\frac{n}{p}+h^2\left(1+\frac{n}{p}\left((h^2)^2-2h^2\right)\right)\\
&= 1+h^2\left(-1+(1-h^2)h^2\frac{n}{p}+1+\frac{n}{p}\left((h^2)^2-2h^2\right)\right)\\
&= 1-\frac{n}{p}(h^2)^2.
\end{aligned}$$

We now consider the case $n>p$. Here one the other hand we can reasonably suppose that $\mathbf{Z}^T\mathbf{Z}\simeq n\mathbf{I}_p$ since we assume the genotypes to be independent and again because of the normalization of $\mathbf{Z}$. Using this approximation one can write

$$\mathbf{Z}^T\mathbf{Z}\simeq n\mathbf{I}_p \Rightarrow \mathbf{K}_\lambda \simeq \frac{1}{n+\lambda}\mathbf{Z}^T$$

First noticing the following algebra

$$\frac{n}{n+\lambda}=\frac{n}{n+p\frac{1-h^2}{h^2}}=\frac{\frac{n}{p}}{\frac{n}{p}+\frac{1-h^2}{h^2}}=\frac{\frac{n}{p}\times h^2}{n/p\times h^2+(1-h^2)}=\frac{\frac{n}{p}\times h^2}{1+h^2\times(\frac{n}{p}-1)}$$

$$\frac{\lambda}{n+\lambda} = \frac{p\frac{1-h^2}{h^2}}{n+p\frac{1-h^2}{h^2}} = \frac{1-h^2}{\frac{n}{p}\times h^2 + (1-h^2)} = \frac{1-h^2}{1+h^2(\frac{n}{p}-1)}$$

and replacing $\mathbf{Z}^T\mathbf{Z}$ by $n\mathbf{I}_p$ we now have

$$\begin{aligned}
\mathbb{E}_{z_{te}}\left[\text{var}(\hat{y}_{te}|z_{te})\right] &= \sigma^2 \text{tr}\left(\mathbf{K}_\lambda \mathbf{K}_\lambda^T\right) \\
&\simeq \sigma^2 \text{tr}\left(\left(\frac{1}{n+\lambda}\right)^2 \mathbf{Z}^T\mathbf{Z}\right) \\
&\simeq \sigma^2 \left(\frac{1}{n+\lambda}\right)^2 np \\
&= \sigma^2 \frac{1}{\frac{n}{p}} \left(\frac{\frac{n}{p}\times h^2}{1+h^2\times(\frac{n}{p}-1)}\right)^2
\end{aligned}$$

$$\begin{aligned}
\mathbb{E}_{z_{te}}\left[\left(\mathbb{E}_{\mathbf{y}_{tr}|z_{te}}\left[\hat{y}_{te}\right] - \mathbb{E}_{y_{te}|z_{te}}[y_{te}]\right)^2\right] &= u^T\left(\mathbf{K}_\lambda\mathbf{Z}\mathbf{K}_\lambda\mathbf{Z} - 2\mathbf{K}_\lambda\mathbf{Z} + \mathbf{I}_p\right)u \\
&\simeq u^T\left(\left(\frac{1}{n+\lambda}\right)^2 \mathbf{Z}^T\mathbf{Z}\mathbf{Z}^T\mathbf{Z} - 2\left(\frac{1}{n+\lambda}\right)\mathbf{Z}^T\mathbf{Z} + \mathbf{I}_p\right)u \\
&\simeq \left(\frac{n}{n+\lambda} - 1\right)^2 u^Tu \simeq \left(\frac{n}{n+\lambda} - 1\right)^2 h^2 \\
&= \left(\frac{1-h^2}{1+h^2(\frac{n}{p}-1)}\right)^2 h^2
\end{aligned}$$

Summing all those expressions, we end up with

$$\mathbb{E}_{\mathbf{y}_{tr},y_{te},z_{te}}\left[(y_{te}-\hat{y}_{te})^2\right] \simeq (1-h^2)\frac{1+\frac{n}{p}h^2}{1+h^2(\frac{n}{p}-1)}$$

In the end we have

$$\mathbb{E}_{\mathbf{y}_{tr},y_{te},z_{te}}\left[(y_{te}-\hat{y}_{te})^2\right] \simeq \begin{cases} 1-\frac{n}{p}(h^2)^2 \text{ if } n<p \\ (1-h^2)\frac{1+\frac{n}{p}h^2}{1+h^2(\frac{n}{p}-1)} \text{ else,} \end{cases}$$

$$\mathbb{E}_{z_{te}}\left[\text{var}(\hat{y}_{te}|z_{te})\right] \simeq \begin{cases} (1-h^2)(h^2)^2\frac{n}{p} \text{ if } n<p \\ (1-h^2)\frac{1}{\frac{n}{p}}\left(\frac{\frac{n}{p}\times h^2}{1+h^2\times(\frac{n}{p}-1)}\right)^2 \text{ else,} \end{cases}$$

$$\mathbb{E}_{z_{te}}\left[\left(\mathbb{E}_{\mathbf{y}_{tr}|z_{te}}\left[\hat{y}_{te}\right]-\mathbb{E}_{y_{te}|z_{te}}[y_{te}]\right)^2\right] \simeq \begin{cases} h^2\left(1+\frac{n}{p}\left((h^2)^2-2h^2\right)\right) \text{ if } n<p \\ \left(\frac{1-h^2}{1+h^2(\frac{n}{p}-1)}\right)^2 h^2 \text{ else.} \end{cases}$$

Graphs of the theoretical squared bias and the variance with respect to the $\log(\frac{n}{p})$ are plotted respectively in S3 and S4.

8.2 Approximation of the mean squared error on the training set

We quickly remind our approximations

$$\mathbf{H}_\lambda = \mathbf{Z}\mathbf{K}_\lambda \simeq \begin{cases} \frac{1}{p+\lambda}\mathbf{Z}\mathbf{Z}^T \simeq \frac{p}{p+\lambda}\mathbf{I}_n \simeq h^2\mathbf{I}_n \text{ if } n<p \\ \frac{1}{n+\lambda}\mathbf{Z}\mathbf{Z}^T \text{ otherwise.} \end{cases}$$

Assuming $\mathbf{Z}$ is fixed and writing the expectation over $\mathbf{y}_{tr}$ of the mean squared error on the training set, we have

$$\begin{aligned} \mathbb{E}_{\mathbf{y}_{tr}}\left[\frac{1}{n}(\mathbf{y}_{tr}-\hat{\mathbf{y}}_{tr})^T(\mathbf{y}_{tr}-\hat{\mathbf{y}}_{tr})\right] &= \mathbb{E}_{\mathbf{y}_{tr}}\left[\frac{1}{n}\mathbf{y}_{tr}^T(\mathbf{I}_n-\mathbf{H}_\lambda)^2\mathbf{y}_{tr}\right] \\ &= \frac{1}{n}\left(\text{tr}((\mathbf{I}_n-\mathbf{H}_\lambda)^2\times\sigma^2\mathbf{I}_n)+(\mathbf{Z}u)^T(\mathbf{I}_n-\mathbf{H}_\lambda)^2(\mathbf{Z}u)\right). \end{aligned}$$

The focus is the approximation of $(\mathbf{I}_n-\mathbf{H}_\lambda)^2$:

$$(\mathbf{I}_n-\mathbf{H}_\lambda)^2 \simeq \begin{cases} (1-h^2)^2\mathbf{I}_n \text{ if } n<p \\ \mathbf{I}_n-2\times\frac{1}{n+\lambda}\mathbf{Z}\mathbf{Z}^T+\left(\frac{1}{n+\lambda}\right)^2\mathbf{Z}\mathbf{Z}^T\mathbf{Z}\mathbf{Z}^T \simeq \mathbf{I}_n-\frac{2}{n+\lambda}\mathbf{Z}\mathbf{Z}^T+\frac{n}{(n+\lambda)^2}\mathbf{Z}\mathbf{Z}^T \text{ else.} \end{cases}$$

The approximation is straightforward for $n<p$. We thus focus on the $n>p$ case.

$$\text{tr}\left(\mathbf{I}_n - \frac{2}{n+\lambda}\mathbf{Z}\mathbf{Z}^T + \frac{n}{(n+\lambda)^2}\mathbf{Z}\mathbf{Z}^T\right) = n - \frac{2}{n+\lambda}\text{tr}(\mathbf{Z}\mathbf{Z}^T) + \frac{n}{(n+\lambda)^2}\text{tr}(\mathbf{Z}\mathbf{Z}^T)$$
$$\simeq n - 2p\frac{n}{n+\lambda} + p\left(\frac{n}{n+\lambda}\right)^2$$

$$(\mathbf{Z}u)^T(\mathbf{Z}u) \simeq nh^2$$
$$(\mathbf{Z}u)^T(-\frac{2}{n+\lambda}\mathbf{Z}\mathbf{Z}^T)(\mathbf{Z}u) = -\frac{2}{n+\lambda}u^T\mathbf{Z}^T\mathbf{Z}\mathbf{Z}^T\mathbf{Z}u \simeq -2n\frac{n}{n+\lambda}u^Tu \simeq -2n\frac{n}{n+\lambda}h^2$$
$$(\mathbf{Z}u)^T(\frac{n}{(n+\lambda)^2}\mathbf{Z}\mathbf{Z}^T)(\mathbf{Z}u) = \frac{n}{(n+\lambda)^2}u^T\mathbf{Z}^T\mathbf{Z}\mathbf{Z}^T\mathbf{Z}u \simeq n\left(\frac{n}{n+\lambda}\right)^2 h^2.$$

Factorizing those results according to $\frac{n}{n+\lambda}$ and $\left(\frac{n}{n+\lambda}\right)^2$ and using the algebras described above we end up with

$$\mathbb{E}_{\mathbf{y}_{tr}}\left[\frac{1}{n}(\mathbf{y}_{tr} - \hat{\mathbf{y}}_{tr})^T(\mathbf{y}_{tr} - \hat{\mathbf{y}}_{tr})\right] \simeq \begin{cases} (1-h^2)^2 \text{ if } n < p \\ 1 - 2\frac{n}{n+\lambda}\left(\frac{p}{n}(1-h^2) + h^2\right) + \left(\frac{n}{n+\lambda}\right)^2\left(\frac{p}{n}(1-h^2)+h^2\right) \text{ otherwise.} \end{cases}$$

Graph of the theoretical mean squared error on the training set with respect to the $\log(\frac{n}{p})$ is plotted in S5.

8.3 Approximation of the squared correlation on the test set

Here we will explain our approximation of the correlation between the phenotype and the prediction. Assuming $\mathbf{Z}$ is fixed, the correlation is

$$\text{corr}(\hat{y}_{te}, y_{te}) = \frac{\text{cov}_{\mathbf{y}_{tr},y_{te},z_{te}}(y_{te}, \hat{y}_{te})}{\sqrt{\text{var}_{\mathbf{y}_{tr},y_{te},z_{te}}[y_{te}]}\sqrt{\text{var}_{\mathbf{y}_{tr},y_{te},z_{te}}[\hat{y}_{te}]}}.$$

Estimating each of those 3 terms:

$$\begin{aligned}\text{var}_{\mathbf{y}_{tr},y_{te},z_{te}}[\hat{y}_{te}] &= \mathbb{E}_{\mathbf{y}_{tr},y_{te},z_{te}}[\hat{y}_{te}^2] - \mathbb{E}_{\mathbf{y}_{tr},y_{te},z_{te}}[\hat{y}_{te}]^2 \\ &= \mathbb{E}_{\mathbf{y}_{tr},y_{te},z_{te}}[z_{te}^T\mathbf{K}_\lambda\mathbf{y}_{tr}\mathbf{y}_{tr}^T\mathbf{K}_\lambda^T z_{te}] - \mathbb{E}_{\mathbf{y}_{tr},y_{te},z_{te}}[z_{te}^T\mathbf{K}_\lambda\mathbf{y}_{tr}]^2 \\ &= \mathbb{E}_{\mathbf{y}_{tr}}\mathbb{E}_{y_{te},z_{te}|\mathbf{y}_{tr}}[z_{te}^T\mathbf{K}_\lambda\mathbf{y}_{tr}\mathbf{y}_{tr}^T\mathbf{K}_\lambda^T z_{te}] - \mathbb{E}_{\mathbf{y}_{tr}}\mathbb{E}_{y_{te},z_{te}|\mathbf{y}_{tr}}[z_{te}^T\mathbf{K}_\lambda\mathbf{y}_{tr}]^2 \\ &= \mathbb{E}_{\mathbf{y}_{tr}}[\text{tr}(\mathbf{K}_\lambda\mathbf{y}_{tr}\mathbf{y}_{tr}^T\mathbf{K}_\lambda^T) + 0] - 0 \\ &= \mathbb{E}_{\mathbf{y}_{tr}}[\mathbf{y}_{tr}^T\mathbf{K}_\lambda^T\mathbf{K}_\lambda\mathbf{y}_{tr}] \\ &= \text{tr}(\mathbf{K}_\lambda^T\mathbf{K}_\lambda \times \sigma^2\mathbf{I}_n) + (\mathbf{Z}u)^T\mathbf{K}_\lambda^T\mathbf{K}_\lambda(\mathbf{Z}u)\end{aligned}$$

$$\begin{aligned}
\mathrm{cov}_{\mathbf{y}_{tr},y_{te},z_{te}}(y_{te},\hat{y}_{te}) &= \mathbb{E}_{\mathbf{y}_{tr},y_{te},z_{te}}[(y_{te}-\mathbb{E}_{\mathbf{y}_{tr},y_{te},z_{te}}[y_{te}])(\hat{y}_{te}-\mathbb{E}_{\mathbf{y}_{tr},y_{te},z_{te}}[\hat{y}_{te}])] \\
&= \mathbb{E}_{\mathbf{y}_{tr},y_{te},z_{te}}[(y_{te}-\mathbb{E}_{\mathbf{y}_{tr},y_{te},z_{te}}[z_{te}^T u + e_{te}])(\hat{y}_{te}-\mathbb{E}_{\mathbf{y}_{tr},y_{te},z_{te}}[z_{te}^T \mathbf{K}_\lambda \mathbf{y}_{tr}])] \\
&= \mathbb{E}_{\mathbf{y}_{tr},y_{te},z_{te}}[(y_{te}\hat{y}_{te})] \\
&= \mathbb{E}_{y_{te},z_{te}}\mathbb{E}_{\mathbf{y}_{tr}|y_{te},z_{te}}[y_{te} z_{te}^T \mathbf{K}_\lambda \mathbf{y}_{tr}] \\
&= \mathbb{E}_{y_{te},z_{te}}[y_{te} z_{te}^T \mathbf{K}_\lambda \mathbf{Z} u] \\
&= \mathbb{E}_{y_{te},z_{te}}[z_{te}^T \mathbf{K}_\lambda \mathbf{Z} u (u^T z_{te} + e_{te}^T)] \\
&= \mathbb{E}_{y_{te},z_{te}}[z_{te}^T \mathbf{K}_\lambda \mathbf{Z} u u^T z_{te}] + \mathbb{E}_{y_{te},z_{te}}[z_{te}^T \mathbf{K}_\lambda \mathbf{Z} u \times e_{te}^T] \\
&= \mathrm{tr}(\mathbf{K}_\lambda \mathbf{Z} u u^T) + 0 + 0 \ \left(z_{te}^T \perp e_{te},\ \mathbb{E}[e_{te}] = 0 \right) \\
&= u^T \mathbf{K}_\lambda \mathbf{Z} u
\end{aligned}$$

$$\begin{aligned}
\mathrm{var}_{\mathbf{y}_{tr},y_{te},z_{te}}[y_{te}] &= \mathbb{E}_{\mathbf{y}_{tr},y_{te},z_{te}}[y_{te}^2] - \mathbb{E}_{\mathbf{y}_{tr},y_{te},z_{te}}[y_{te}]^2 \\
&= \mathbb{E}_{y_{te},z_{te}}[(z_{te}^T u + e_{te})^2] - 0 \\
&= \mathbb{E}_{e_{te},z_{te}}\left[(z_{te}^T u)^2\right] + \mathbb{E}_{e_{te},z_{te}}\left[(e_{te})^2\right] + 2\mathbb{E}_{e_{te},z_{te}}\left[(z_{te}^T u) e_{te}\right] \\
&= \mathbb{E}_{z_{te}}[z_{te}^T u u^T z_{te}] + \mathbb{E}_{e_{te}}[e_{te}^2] + 2\mathbb{E}_{z_{te}}[z_{te}^T u]\mathbb{E}_{e_{te}}[e_{te}] \\
&= u^T u + \sigma^2 + 0
\end{aligned}$$

We replace the empirical covariance matrices by their respective approximation according to the cases $n < p$ and $n > p$.

The $n < p$ case :

$$\mathbf{Z}\mathbf{Z}^T \simeq p\mathbf{I}_n \Rightarrow \mathbf{K}_\lambda \simeq \frac{1}{p+\lambda}\mathbf{Z}^T \Rightarrow \mathbf{K}_\lambda^T \mathbf{K}_\lambda \simeq \frac{(h^2)^2}{p}\mathbf{I}_n$$

$$\begin{aligned}
&\sigma^2 \times \mathrm{tr}(\mathbf{K}_\lambda^T \mathbf{K}_\lambda) \simeq \frac{n}{p}(h^2)^2(1-h^2) \\
&(\mathbf{Z}u)^T \mathbf{K}_\lambda^T \mathbf{K}_\lambda (\mathbf{Z}u) \simeq \frac{(h^2)^2}{p}(\mathbf{Z}u)^T(\mathbf{Z}u) \simeq \frac{n}{p}(h^2)^2 \times h^2 \\
&u^T \mathbf{K}_\lambda \mathbf{Z} u \simeq u^T \frac{1}{p+\lambda}\mathbf{Z}^T \mathbf{Z} u \simeq \frac{1}{p+\lambda} n h^2 = \frac{n}{p}(h^2)^2
\end{aligned}$$

The $n > p$ scenario :

$$\mathbf{Z}^T\mathbf{Z} \simeq n\mathbf{I}_p \Rightarrow \mathbf{K}_\lambda \simeq \frac{1}{n+\lambda}\mathbf{Z}^T \Rightarrow \mathbf{K}_\lambda^T\mathbf{K}_\lambda \simeq \left(\frac{1}{n+\lambda}\right)^2 \mathbf{Z}\mathbf{Z}^T$$

$$\mathrm{tr}(\mathbf{K}_\lambda^T\mathbf{K}_\lambda \times \sigma^2\mathbf{I}_n) \simeq (1-h^2)\left(\frac{1}{n+\lambda}\right)^2 \mathrm{tr}(\mathbf{Z}\mathbf{Z}^T) \simeq (1-h^2)\frac{n}{(n+\lambda)^2}p = (1-h^2)\left(\frac{n}{n+\lambda}\right)^2 \frac{p}{n}$$

$$(\mathbf{Z}u)^T\mathbf{K}_\lambda^T\mathbf{K}_\lambda(\mathbf{Z}u) \simeq \frac{1}{(n+\lambda)^2}u^T\mathbf{Z}^T\mathbf{Z}\mathbf{Z}^T\mathbf{Z}u \simeq \left(\frac{n}{n+\lambda}\right)^2 u^Tu \simeq \left(\frac{n}{n+\lambda}\right)^2 h^2$$

$$u^T\mathbf{K}_\lambda\mathbf{Z}u \simeq \frac{n}{n+\lambda}h^2$$

Concatenating those expressions, we eventually get:

$$\mathrm{corr}(\hat{y}_{te}, y_{te}) \simeq \begin{cases} \frac{\frac{n}{p}(h^2)^2}{\sqrt{\frac{n}{p}(h^2)^2}\sqrt{1}} = \sqrt{\frac{n}{p}}h^2 \text{ if } n < p \\ \frac{\frac{n}{n+\lambda}h^2}{\sqrt{\left(\frac{n}{n+\lambda}\right)^2\left(\frac{p}{n}(1-h^2)+h^2\right)}\sqrt{1}} = \frac{h^2}{\sqrt{\frac{p}{n}(1-h^2)+h^2}}, & \text{otherwise.} \end{cases} \tag{S9}$$

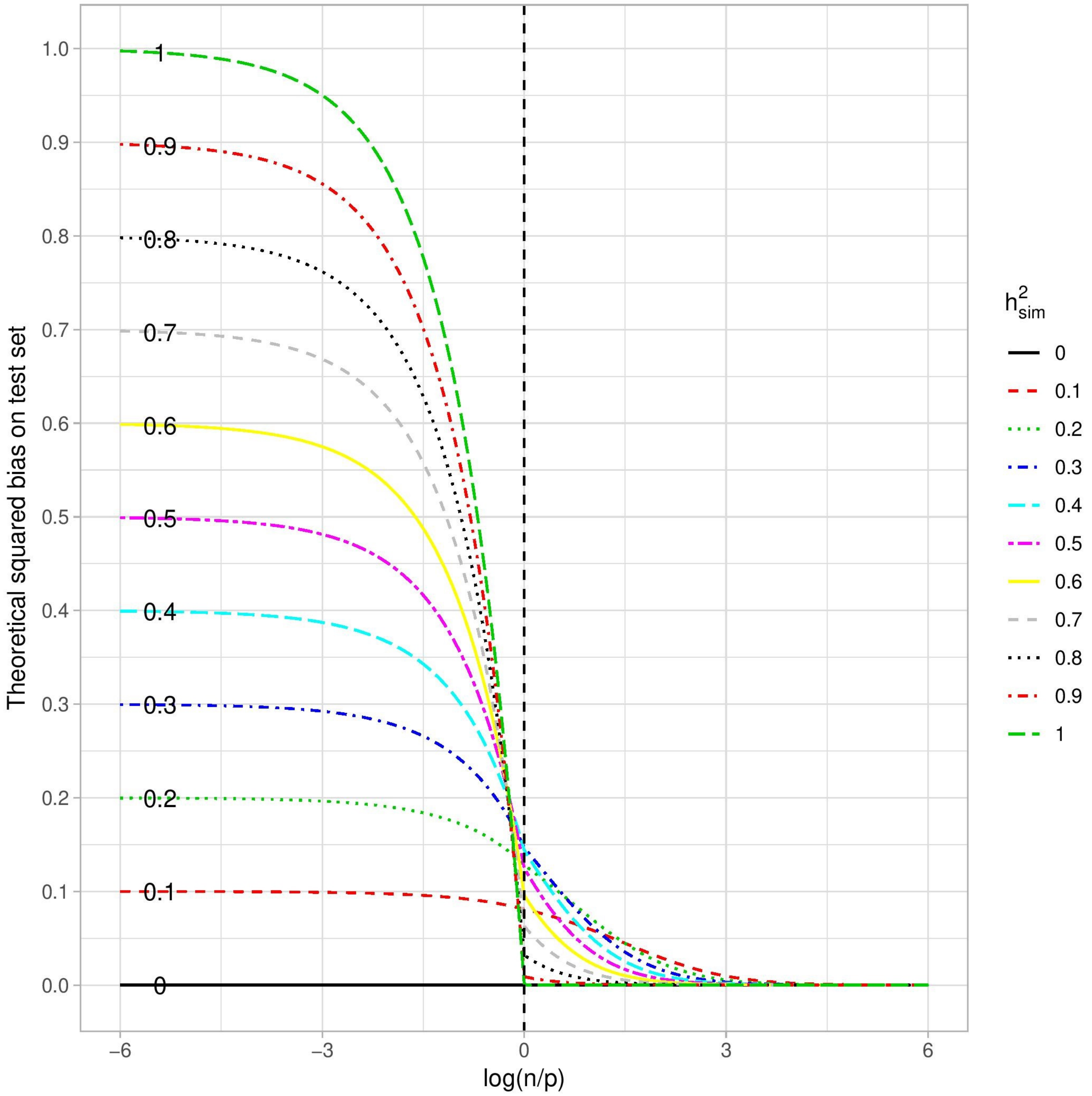

Figure S3: Theoretical squared bias with respect to the log ratio of the number of individuals over the number of variants in the training set. Each curve corresponds to a given heritability (in the narrow sense). Note that the total variance is assumed to be 1.

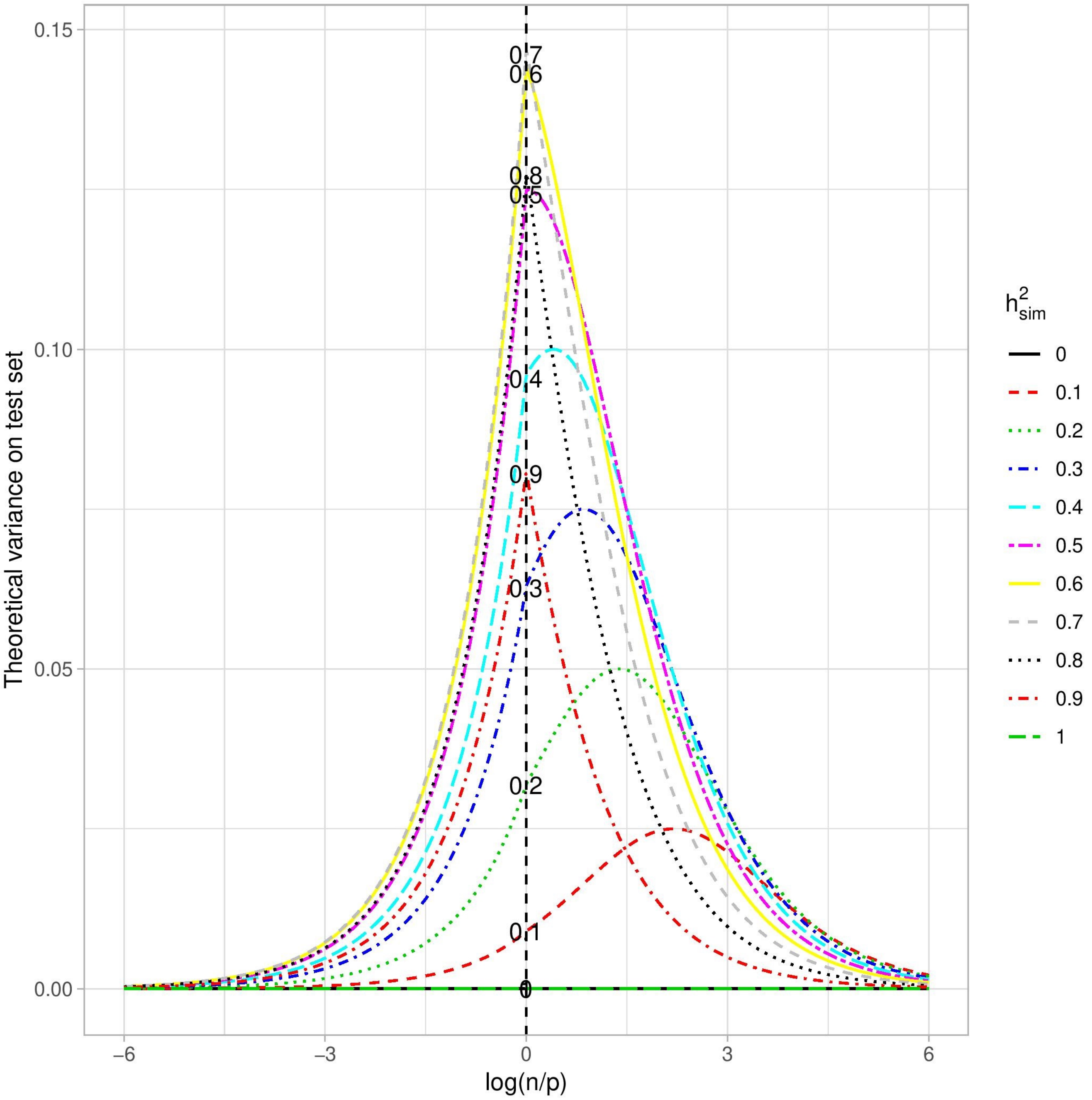

Figure S4: Theoretical variance with respect to the log ratio of the number of individuals over the number of variants in the training set. Each curve corresponds to a given heritability (in the narrow sense). Note that the total variance is assumed to be 1.

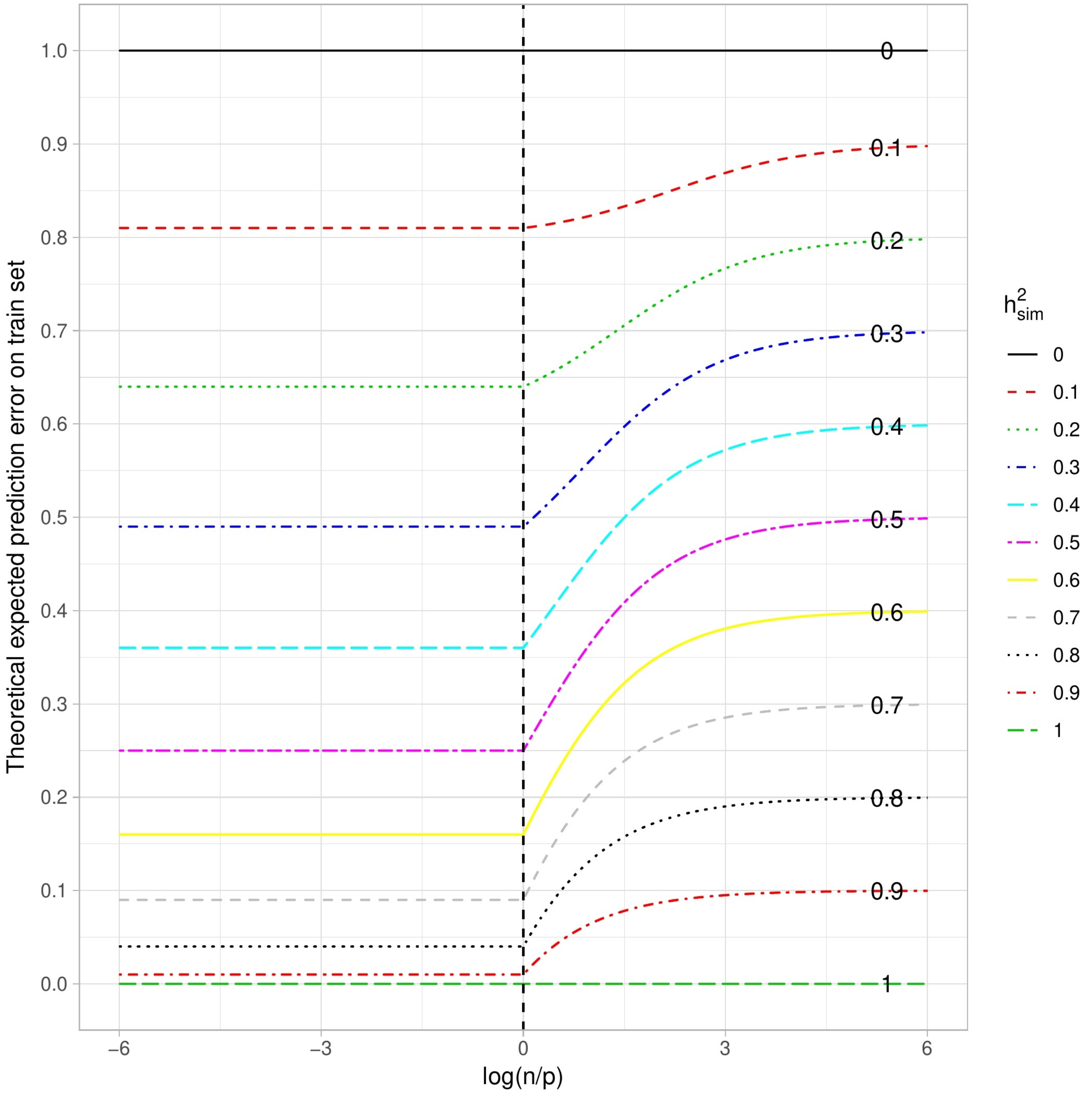

Figure S5: Theoretical quadratic error on the training set with respect to the log ratio of the number of individuals over the number of variants in the training set. Each curve corresponds to a given heritability (in the narrow sense). Note that the total variance is assumed to be 1.
